# Spin Crossover and Exchange Effects on Oxygen Evolution Reaction Catalyzed by Bimetallic Metal Organic Frameworks

**DOI:** 10.1021/acscatal.4c01091

**Published:** 2024-05-20

**Authors:** Guangsheng Liu, Feng Xie, Xu Cai, Jingyun Ye

**Affiliations:** † Department of Chemistry and Biochemistry, Duquesne University, Pittsburgh, Pennsylvania 15282, United States; ‡ Department of Chemistry and Chemical Biology, Rutgers University, Piscataway, New Jersey 08854, United States; § State Key Laboratory of Photocatalysis on Energy and Environment, College of Chemistry, Fuzhou University, Fuzhou 350108, PR China

**Keywords:** bimetallic metal organic framework, oxygen evolution reaction, spin crossover, exchange interaction, pH effect, density functional theory

## Abstract

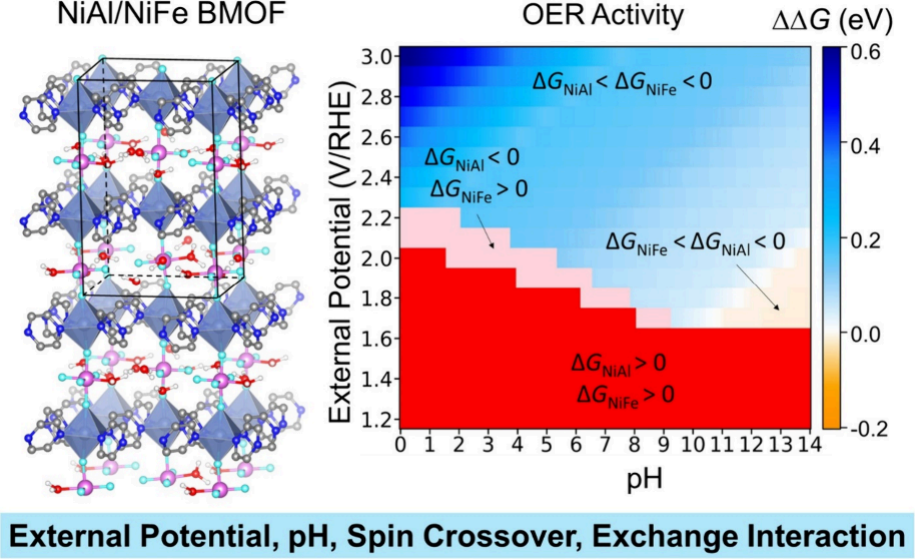

Bimetallic metal–organic frameworks (BMOFs) have shown a superior oxygen evolution reaction (OER) performance, attributed to the synergistic effects of dual metal sites. However, the significant role of these dual-metal synergies in the OER is not yet fully understood. In this study, we employed density functional theory to systematically investigate the OER performance of NiAl- and NiFe-based BMOFs by examining all possible spin states of each intermediate across diverse external potentials and pH environments. We found that the spin state featuring a shallow hole trap state and Ni ions with a higher oxidation state serve as strong oxidizing agents, promoting the OER. An external potential-induced spin crossover was observed in each intermediate, resulting in significant changes in the overall reaction and activation energies due to altered energy levels. Combining the constant potential method and the electrochemical nudged elastic band method, we mapped the minimum free energy barriers of the OER under varied external potential and pH by considering the spin crossover effect for both NiAl and NiFe BMOFs. The results showed that NiFe exhibits better OER thermodynamics and kinetics, which is in good agreement with experimentally measured OER polarization curves and Tafel plots. Moreover, we found that the improved OER kinetics of NiFe not only is attributed to lower barriers but also is a result of improved electrical conductivity arising from the synergistic effects of Ni–Fe dual-metal sites. Specifically, replacing the second metal Al with Fe leads to two significant outcomes: a reduction in both the band gap and the effective hole mass compared to NiAl, and the initiation of super- and double-exchange interactions within the Ni–F–Fe chain, thereby enhancing electron transfer and hopping and leading to the improved OER kinetics.

## 1 Introduction

Electrochemical water splitting is a promising technology for producing hydrogen, a carbon-neutral and energy-rich fuel that offers an alternative to traditional fossil fuels.^1,2^ Nevertheless, electrochemical water splitting currently constitutes a mere 3–5% of the overall industrial hydrogen production. One major obstacle that impedes electrochemical water splitting from being widely utilized is the sluggish kinetics associated with the oxygen evolution reaction (OER), a process involving four proton-coupled electron transfer (PCET) steps.^3^ Although Ir- and Ru-based compounds have exhibited remarkable OER activity, their high cost and scarcity limit their application on a commercial scale.^4−8^ To overcome these limits, extensive efforts have been devoted to developing low-cost, highly active, and durable OER catalysts. Bimetallic catalysts, particularly those based on first-row transition metals (such as Fe, Co, Ni), show superior electrochemical performance due to synergistic effects between mixed metal sites, compared to single-metal catalysts.^9−14^ Understanding the reaction mechanism, active sites, and these synergistic effects is of great importance for developing effective bimetallic OER catalysts.

Metal–organic frameworks (MOFs) are porous crystalline materials constructed by the coordination of organic linkers and metal ions/clusters, which have received significant interest owing to their ultrahigh porosity, large surface area, well-defined structure, remarkable tunability, and diverse functionalities.^15^ Previous MOF research primarily focused on single-metal frameworks. However, a new trend is emerging: the synthesis of functional MOFs with two different metal ions, bimetallic metal–organic frameworks (BMOFs) that offer a diverse range of superior properties in comparison to single-metal MOFs.^16−21^ BMOFs have emerged as intriguing catalysts for OER, driven not only by their diverse advantageous MOF properties but also critically by offering an innovative platform to optimize synergistic effects between mixed-metal atoms, enhancing the intrinsic properties of MOFs, including the coupling effect between metals, improved conductivity, and enhanced charge transport, leading to superior catalytic performance compared to monometallic MOFs.^15,22−28^

Introducing missing linker defects in ultrathinning BMOFs has been shown to enhance OER performance, particularly due to the coupling effect of dual open-metal sites.^29,30^ For instance, Zhao and co-workers^29^ synthesized ultrathin NiCo bimetal–organic framework nanosheets (NiCo-UMOFNs) with a uniform thickness of ∼3.1 nm. The NiCo-UMOFNs exhibited excellent performance as a promising electrocatalyst in the OER, with an overpotential of 189 mV at a current density of 10 mA·cm^–2^ when the MOF nanosheets were loaded on copper foam. The excellent OER activity of NiCo-UMOFNs is attributed to the strong coupling interaction between Co and Ni and the presence of unsaturated metal centers as active sites, as evidenced by X-ray spectroscopy and DFT calculations. Other studies have also explored the coupling effect in different BMOFs for OER. Hai and co-workers^30^ reported the observation of the coupling effect of Ni and Fe in NiFe bimetal ultrathin MOF nanosheets (NiFe-UMNs) contributing to efficient OER performance. Zhao and co-workers^31^ investigated a series of Ni_*x*_Co_*y*_-MOF-74 and Ni_*x*_Fe_*y*_-MOF-74 nanosheets for OER, highlighting the low overpotential of 198 mV at 10 mA cm^–2^ of the Ni_0.9_Fe_0.1_-MOF-74 electrocatalyst.

Incorporating conductive and magnetic organic linkers is a highly effective approach for improving the conductivity of MOFs, subsequently enhancing OER kinetics.^32,33^ For instance, Liu et al.^34^ reported a Co-based MOF (Co-tzpa) with a lower charge transfer resistance demonstrating improved OER kinetics, surpassing Co_3_O_4_ or CoOOH, due to the introduction of tetrazolate as linkers. Similarly, Adpakpang et al.^35^ synthesized a Co-triazole MOF (Co-trz) with a low overpotential and good kinetics for OER, attributed to the increased electrical conductivity. Furthermore, nitrogen-containing organic ligands like^36,37^ have shown promise in modifying the magnetic properties of MOFs, thereby enhancing OER activity through π–*d* interactions between the nitrogen-containing organic ligand and the metal center.^33,38^

**Figure 1:**
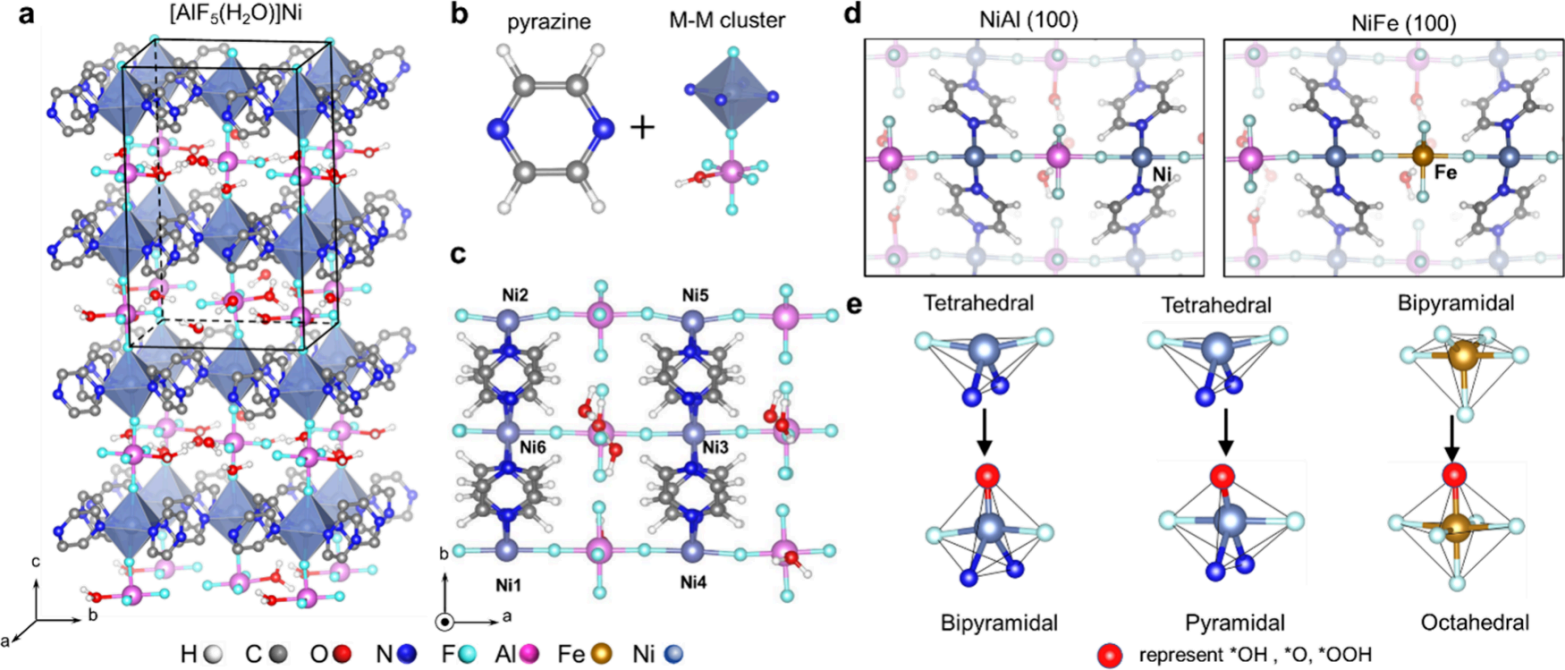
Structure of AlFFIVE-1-Ni: (a) side view, (b) building blocks including pyrazine linker and bimetallic inorganic node (Ni–Al or Ni–Fe). (c) Side-view of the NiAl(100) slab model with lattice constants of *a* = 9.736 Å, *b* = 15.536 Å, *c* = 13.481 Å; α = β = 90.00° γ = 90.45° separated by a 15 Å vacuum. (d) Top view of NiAl (100) and NiFe (100) slab models. (e) Local coordination geometries of Ni and Fe sites before and after the adsorption of intermediate species.

The understanding of the pivotal role played by synergistic bimetallic catalysts in the OER remains limited, yet it is imperative for advancing novel BMOFs. Recently, Cadiau and co-workers^39^ reported two hydrolytically stable fluorinated bimetallic metal–organic frameworks, AlFFIVE-1-Ni and FeFFIVE-1-Ni, abbreviated as NiAl and NiFe, respectively, which could be promising bimetallic electrocatalysts for OER. NiM (M = Al or Fe), represented in Figure 1a, features a cubic topology with a chemical formula NiMF_5_(H_2_O)(pyr)_2_·2(H_2_O), constructed by the pillaring of Ni(II)-pyrazine 2-periodic square-grid layers (Figure 1c) cross-linked by [MF_5_(H_2_O)]^2–^ inorganic pillars (Figure 1b). In this work, we employed DFT to investigate the effect of spin crossover and super/double exchange interactions induced by the dual transition metal sites with multivalent oxidation states and spin states on the OER activity of NiAl and NiFe under varying external potentials at different pH levels. Specifically, we systematically investigated all possible spin states for each of the OER intermediates on both NiAl(100) and NiFe(100) surfaces. We observed the spin crossover induced by the external potential, and subsequently, we explored the effects of spin crossover, exchange interactions, and pH on the OER activity. Our predictions indicate that NiFe exhibits superior OER activity and kinetics compared to NiAl, aligning with experimental OER polarization curves and Tafel plots. We conducted comprehensive electronic structure analyses to elucidate the enhanced OER performance of NiFe.

## 2 Computational Models and Methods

Spin-polarized Kohn–Sham DFT calculations were performed to optimize the unit cell and surface slabs using VASP 6.3.2.^40,41^ with a plane wave basis set and the projector augmented wave (PAW) potentials.^42^ Electronic exchange and correlation were described using the Perdew–Burke–Ernzerhof functional^43^ with Grimmes’ D3 correction.^44^ Standard PAW potentials were used with 1s for H; 2s and 2p for C, O, N, and F; 3d and 4s for Ni; and 3d for Fe were being treated as the valence state. The kinetic energy cutoff of 600 eV with a 1 × 1 × 1 Γ-point grid was used for unit cell optimization. The PBE-computed lattice constants for NiAl and NiFe unit cells agree with the experimental data with errors less than 2% (Table S1). To examine the OER activity, we selected the (100) facet, as each Ni ion at the top layer has four coordinates, which provides an additional unsaturated site compared to Ni ions on other facets, potentially enhancing its reactivity (Figure S1). The NiAl(100) surface slab model (Figure 1c) was constructed based on the optimized unit cell, which consists of three metal layers cross-linked by two pyrazine linker layers. NiFe(100) was created by replacing one Al atom at the top layer of NiAl(100) (Figure 1d). Additionally, the stability and magnetic moment fluctuation of NiAl(100) were investigated via *Ab Initio* Molecular Dynamics (AIMD) simulation under NVT ensemble (Figure S2) at a time step of 0.50 fs with Nosé–Hoover thermostats at 300 K.^45,46^ The results indicate that NiAl(100) remains stable at 300 K and spin flip can occur on Ni ions leading to singlet, doublet, and triplet states. Hence, our investigation delved into the reaction mechanisms by considering all possible spin states for surface (*) and each intermediate, *OH, *O, and *OOH. The NiAl(100) surface model comprises six Ni ions, offering 13 possible spin states. These include a low-spin (LS) state with a total magnetic moment of 0 μ_B_, a high-spin (HS) state with a moment of 12 μ_B_, and intermediate-spin (IS) states with moments ranging from 1 to 11 μ_B_. For the NiFe(100) surface, 18 spin states were considered. This is attributed to the Fe ion, which can have a magnetic moment varying between 0 and 5 μ_B_. Consequently, a total of 13 and 18 spin states were calculated for the NiAl(100) and NiFe(100) surfaces, respectively. The coordination geometry of surface Ni sites (Ni[5] or Ni[2] with two open sites resulting from the two missing pyrazine linkers) could transfer from a tetrahedron geometry in a surface model (*) to either a pyramidal or bipyramidal geometry upon the adsorption of intermediate species (Figure 1e). Given this, we considered two initial structures with all possible spin states. This led to calculations of 26 and 36 magnetic structures for each intermediate for the NiAl and NiFe systems, respectively. Ni[5] is considered the active site for all surface slab calculations. The bottom two metal layers together with one pyrazine linker layer and H_2_O in the pores are fixed, while other atoms are relaxed with dipole correction included due to the asymmetry of the surface model. The surface slab optimization used an energy cutoff of 520 eV with a 1 × 1 × 1 Γ-point grid and 0.05 eV Gaussian electronic smearing width due to the large size of the model (K-point tests are summarized in Figure S3). The structures were relaxed until the force on each atom is less than 0.02 eV/Å, and the energy change is less than 10^–6^ eV.

### Constant Charge Method (CCM)

To describe the strongly correlated systems that have transition metals such as Fe and Ni, self-consistent field (SCF) calculations were performed using the hybrid Heyd–Scuseria–Ernzerh (HSE06) functional^47,48^ with an exact Hatree–Fock (HF) exchange percentage of α = 0.15 to calculate the energies and electronic structures based on the PBE-optimized geometries. We used the reparametrized HSE06 with α = 0.15 in contrast to the standard HSE06 with α = 0.25 or PBE+U because (1) the reparametrized HSE06 with α = 0.15 offers significant improvement over the standard HSE06 or PBE+U for the thermodynamic properties and electronic structure predictions for OER-catalyzed by Ni/Fe systems;^49−51^ (2) PBE+U causes the spurious electron delocalization;^52−54^ and (3) the large variations of *U* values were observed when the Ni ion was in different chemical environments. As shown in Figure S4, the calculated *U* value of Ni[5] using the linear response method is 5.88 eV for the bare surface, which decreased to 3.72 eV after the adsorption of O at Ni[5]. For the CCM, the Gibbs free energy diagram at the potential of zero charge (PZC) was plotted using the corrected HSE energies by including the enthalpy and entropy calculated using the partition function (see Section 6 of the Supporting Information). The Gibbs free energy diagrams calculated using different functionals are compared as summarized in Section 7 of the Supporting Information.

### Constant Potential Method (CPM)

The applied potential to the electrochemical interface was simulated by adding or removing electrons from the surface slabs. The charged surface slabs together with the compensating homogeneous background charge were optimized using constrained DFT (cDFT) with the PBE functional. cDFT offers the possibility to constrain both the direction and magnitude of the magnetic moment for Ni and Fe ions in the surface slab model to track the total energies of the different spin states as a function of the external potential. Subsequently, SCF calculations were performed on the cDFT-optimized structure through VASPsol^55,56^ with a continuum dielectric model. The relative permittivity of 80 was chosen to mimic an aqueous electrolyte environment, setting the TAU to 0 to ignore the influence of cavitation energy.

The potential-dependent grand free energy (Ω) of the electrode can be defined as eq 1:^57^
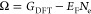
1where *G*_DFT_ is the SCF energy calculated using VASPsol corrected by including the contribution of enthalpy and entropy calculated via partition function, *E*_F_ is the Fermi energy of the Fermi–Dirac distribution on the Kohn–Sham eigenvalues, and *N*_e_ is the excess number of electrons with respect to the uncharged slab.

The electric potential (*U*_Ne_) of the charged slab with *N*_e_ excess electrons with respect to the standard hydrogen electrode (SHE: H_2_/H^+^, pH = 0) at 25 °C can be defined as eq 2.
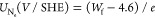
2where *W*_f_ is the work function of the charged surface slab and 4.6 eV is the work function of SHE. To model the charged interfaces at different applied potentials, we optimized each intermediate with a background charge from −2.0 |e^–^| to 2.0 |e^–^| with a step size of 0.5 |e^–^|. The free energy at the 11 charge values were then fitted to a quadratic function, which aligns with a capacitor formed by the charged-slab/background-charge system, as shown by eq 3:
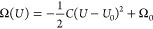
3where *U*_0_ refers to PZC, Ω_0_ is the grand free energy at PZC conditions, and *C* is the capacitance of the surface. The grand free energy of the intermediate at any external potential (*U*) can be calculated using eq 3.

The pH can profoundly affect the adsorption energies of intermediates by changing the electric potential, surface charge, and protonation/deportation of the catalyst surface. Here, we included the effect of pH on the electric potential via eq 4:
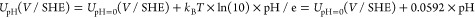
4where *k*_B_ is the Boltzmann constant and *T* is the temperature. *k*_B_*T* × ln(10) = 0.0592, where *T* = 298.15 K. That being noted, we adopted SHE as a reference electrode; one can also use the reversible hydrogen electrode (RHE) as a reference electrode by changing the electric potential following eq 5:
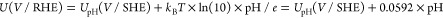
5where *U*(*V*/RHE) is the electric potential of electrode/electrolyte interface with respect to the RHE.

### Transition State Calculation

The electrochemical nudged elastic band (eNEB) method was used to search for the transition state, which enables all of the images along the reaction coordinate under the same potential.^58^ We considered the solvent effects in eNEB calculations by introducing H_9_O_4_^+^ in the proximity of the surface. The initial guesses for eNEB are generated via the image-dependent pair potential method to improve the search efficiency.^58^

### Electronic Structure Calculation

DDEC6 charges are calculated using the Chargemol package.^59^ The crystal orbital Hamiltonian population (COHP) was calculated by the LOBSTER 4.1.0 package for the chemical bonding analysis, which reconstructs the orbital-resolved electronic structure via projection of the PAW wave functions onto atomic-like basis functions by the pbeVASPFit2015 basis set.^60,61^ The DOS of each intermediate for the OER was calculated via the HSE06 functional with α = 0.15. To balance accuracy and efficiency, the HLE17 exchange–correlation functional was used to calculate the density of state (DOS) and band structures of NiAl and NiFe bulk materials.^62,63^

## 3 Results and Discussion

### Energetics, Spin, Geometry of *, *OH, *O, and *OOH

For each intermediate involved in the OER, including *, *OH, *O, and *OOH, we have computationally screened all of the possible initial structures (pyramidal and bipyramidal) and all of the possible spin states. The relative electronic energies, atom-projected magnetic moment, geometry configuration, and total magnetic moment are summarized in Tables S3–S7. The energy of each intermediate primarily relies on the spin state and coordination geometry of Ni ions. First, the energy of the system is dependent on the parity of the total magnetic moment. When the total magnetic moment of the system has the same parity as the number of total valence electrons, the energy of the system is lower; otherwise, the energy is higher. For example, the NiAl(100) surface has an even number of valence electrons; therefore, the NiAl(100) surface with an even total magnetic moment has lower energy compared with the odd ones (see sheet HSE015-AlNi of Energy_data.xlsx). Second, when the parity of the total valence electrons and magnetic moment is the same, the energy of the system is greatly influenced by the spin flip. As shown in Table 1, *OH with a spin state of 222212 obtains the lowest energy at PZC for both NiAl and NiFe. If the spin state of one Ni ion of *OH changes from triplet to singlet, the energy increases by approximately 0.6 eV. For example, we observe energy increases of 0.54 eV from S1 to S3, 0.66 eV from S1 to S4, and 0.56 eV from S4 to S5 in the case of NiAl(100). A similar behavior was observed for NiFe(100), and the energy of S1 increased by 0.54 eV when the spin state of the Ni[3] ion changes from triplet to singlet (S5). Third, the relative energy depends on the coordination geometry and hydrogen bond. Overall, at PZC conditions, the ground states prefer the pyramidal geometry for all the intermediates on both NiAl (100) and NiFe (100). For most of the excited states, the pyramidal geometries have lower energies than the bipyramidal geometries (Figures S8 and S9). Furthermore, the formation of a hydrogen bond could stabilize the structure by approximately 0.1 eV compared to the system without a hydrogen bond. As shown in Table 1, *OH/NiFe with S2 is 0.1 eV lower than that with S3 due to the formation of an O–H···F hydrogen bond (see the structures of S2 and S3 in Figure S10).

**Table 1:** Relative Electronic Energies, Atom-Projected Magnetic Moment *M* (μ_B_), Geometry, and the Total Magnetic Moment *M*_total_ (μ_B_) of *OH at Different Spin States Calculated Using HSE06 with α = 0.15

*OH	NiAl				NiFe			
spin state	Δ*E* (eV)	*M*_Ni[1–6]_	config	*M*_total_	Δ*E* (eV)	*M*_FeNi[1–6]_	config	*M*_total_
S6^d^	1.83	[022222]^c^	BP^a^	4	0.86	[5222202]	P^b^	3
S5	1.22	[002212]	P–H^a^	1	0.54	[5202212]	P–H	4
S4	0.66	[022212]	P–H	5	0.27	[5222222]	BP	12
S3	0.54	[202212]	P–H	3	0.13	[5222212]	P	16
S2	0.48	[222222]	BP	12	0.03	[5222212]	P–H	16
S1	0.00	[222212]	P–H	11	0.00	[5222212]	P–H	6

a BP represents bipyramidal.

b P and P–H represent pyramidal without and with a H···F hydrogen bond, respectively.

c Atom-projected magnetic moment (net atom-projected spins) are assigned to each Ni and Fe ions based on rules provided in Table S3. 0, 1, and 2 represent singlet, doublet, and triplet local spin states, respectively. The numbers without and with underscore represent spin up and spin down, respectively.

d The six selected spin states are the ground state (S1) with lowest energy and the five excited states (S2–S6) including four intermediate-spin states and the spin state with highest energy. When the spin state has degenerate states, the one with lower energies are reported in the table and others are provided in the SI.

### OER Mechanism at PZC

**Figure 2:**
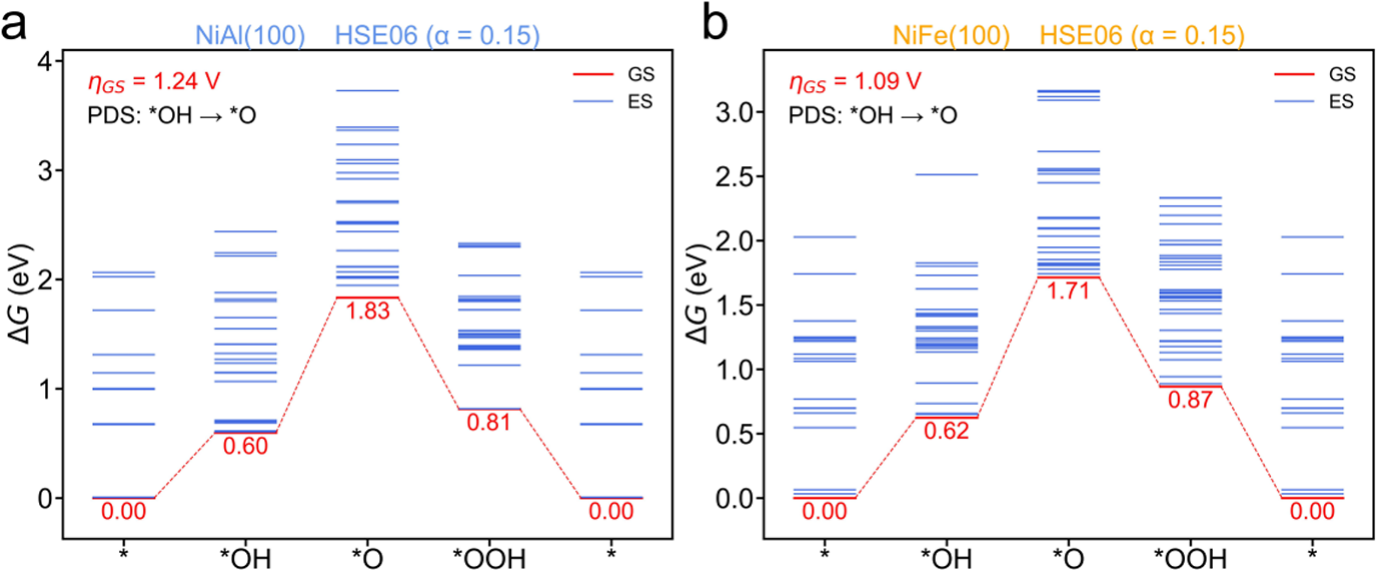
Relative Gibbs free energy diagrams of the OER catalyzed on (a) NiAl (100) and (b) NiFe (100) at the PZCs calculated using the HSE functional with HF exchange of α = 0.15. Red and blue lines represent the ground states and excited states, respectively. The states that have the energy difference less than 0.1 eV are considered degenerate with the lowest state included in this diagram. η_GS_ is the onset potential of the OER with each intermediate at the ground state.

Figure 2 displays the Gibbs free energy diagrams of OER catalyzed by NiAl(100) and NiFe(100) at their PZC. The calculated onset potentials are 1.24 and 1.09 V for NiAl(100) and NiFe(100), respectively, when each intermediate is at its ground state. The potential-determining step (PDS) of OER for both NiAl and NiFe BMOFs are *OH and *O. Potential of zero charge is one of the most fundamental ideas in electrochemistry to define the potential at which the electrode has zero surface charge.^64^

At PZC, the binding energies of intermediates are calculated using a CCM, where the charge of the electrode before and after the adsorption remains constant. However, under the electrocatalysis reaction conditions, the electrode potential is typically held constant, rather than the total charge. In this case, the charge at the electrochemical interface varies before and after the adsorption of intermediate species and changes when the different species are adsorbed. This charge effect was found to have a strong impact on electrochemical reactions, especially for 2D materials.^65^

Spin crossover is commonly observed with first-row transition metal complexes, where the electronic spin state of the metal ion changes due to an external stimulus including temperature, pressure, light, magnetic field, and electric field.^66,67^ In electrocatalysis, the spin crossover effect has rarely been studied. Duan and Henkelman^68^ observed a spin crossover effect induced by the applied potential on the adsorption energies of intermediates. For NiAl/NiFe surfaces, the Ni active sites might undergo spin crossover stimulated by a strong electric field when the external potential is applied.

To provide a more accurate understanding of the effect of external potential, spin crossover, and pH on the energies of intermediates on NiAl/NiFe (100) surfaces, the CPM combined with the cDFT has been employed to study the OER activity of NiAl/NiFe BMOFs.

### Spin Crossover Effect

**Figure 3:**
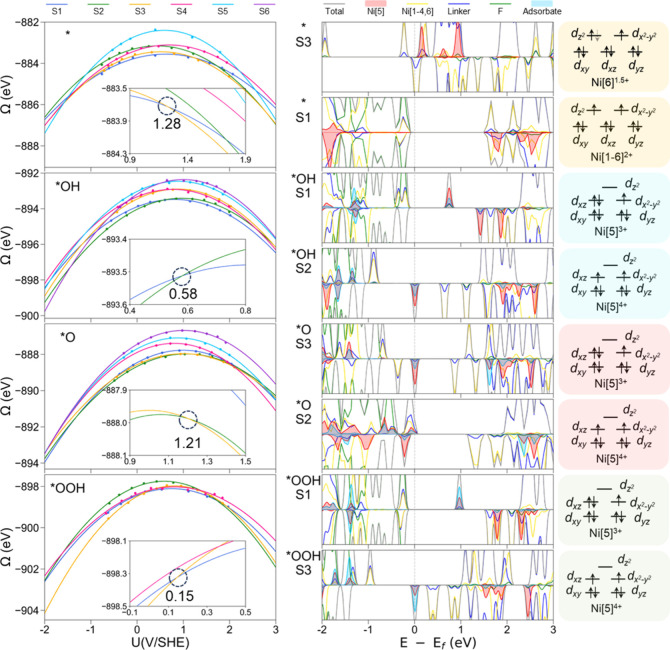
Left panel: calculated total energies (dots) and quadratic function fits (lines) of the selected spin states, spin1–spin6 (S1–S6) of the NiAl(100) surface (*), *OH/NiAl(100), *O/NiFe (100), and *OOH/NiAl (100) as a function of the external potential. The spin crossover points are circled by the dashed line, with the external potential value indicated underneath. Projected density of states (PDOS) (middle panel) and 3d orbital diagrams of Ni ions for each intermediate with the spin states of interest (right panel). The solid arrow and the gray dashed arrow represent the single electron and fractional electron occupation, respectively.

We have investigated the spin crossover effect for the six selected spin states (as shown in Table 1 and Tables S4–S7) of the four intermediates involved in the OER on NiAl(100) and NiFe(100) surfaces without considering the effect of pH, naming the default pH = 0. Figure 3 displays the grand free energies as a function of the external potential of each intermediate for NiAl(100), projected density of state (PDOS) of the lower energy states, and 3d orbital diagrams of Ni ions of interest. The PDOS and plots for NiFe(100) are presented in Figure S11. The potential-dependent grand free energies are well represented by a quadratic function, and the fitted parameters (*U*_0_ and C) are given in Table S8. Our discussion will focus on the spin crossover points for lower energy spin states since they are most likely to occur.

### Surface (*)

Multiple spin crossover points were observed in the potential range of interest for NiAl(100) because different spin states exhibited unique parabolic energy trends with the voltage. Under the positive external potentials, S1 of NiAl(100), initially more stable, becomes less stable than S3 when the external potential is greater than 1.28 V/SHE, and when the external potential is greater than 1.7 V/SHE, it becomes less stable than S2. The spin crossover between S1 and S3 occurs at a potential (1.28 V/SHE) lower than that between S1 and S2 (1.7 V/SHE). This is primarily due to the proximity of the PZC values of S1 (*U*_0_ = 0.62 V/SHE) and S3 (*U*_0_ = 0.51 V/SHE), in contrast to S2 (0.44 V/SHE). Under the negative external potential, the spin crossover for NiAl(100) occurs at around −1.5 V/SHE between S5 and S1, S3, and S4, suggesting that the spin-state transition from the ground state (S1) to excited states (S3, S4, and S5) could be potentially introduced by exerting negative external potential. The excited NiAl surface could be retained rather than relaxed to the ground state due to the hysteresis effect, which might lead to better OER activity than the surface at the ground state.^69,70^ At an external potential less than 1.28 V/SHE, S3, [222221.5] is less stable than S1, [222222], which is mainly due to Ni^2+^ with octahedral coordination geometry, which prefers a triplet rather than fractional occupation, as demonstrated in the 3d orbital diagrams of Ni[6] (right panel of Figure 3). The Fermi energy of S3 shifts to a higher energy level compared to that of S1 as shown in PDOS, and the symmetry of spin-up and spin-down of S3 is broken due to the fractional electron occupation. For NiFe(100), the spin crossover occurs at 2.21 V/SHE between the ground state (S1) and the excited state (S5) while the spin crossover between the S1 [5222222] and S4 [5212222] states appears at −0.5 V/SHE, indicating that a relatively moderate negative external potential can alter the magnetic moment of Ni[2] of NiFe(100).

### *OH

For *OH/NiAl, the potential for spin crossover is located at 0.58 V/SHE between the ground state (S2) and the first excited state (S1). When the external potential falls below 0.58 V/SHE, S2 [222222] is more stable than S1 [222212], which implies that triplet Ni[5] is more stable than doublet Ni[5] at a lower potential when *OH is adsorbed at Ni[5]. From PDOS results, we observed a shallow hole trap state typically found near the Fermi energy level (or close to the valence band maximum) for S2 while a deep hole trap state is located deeper within the band gap and distanced from both the conduction and valence bands for S1.^71,72^ Both shallow and deep hole trap states are composed of Ni[5] and O, suggesting that the shallow and deep holes are localized at Ni[5] and O, respectively. The structure with the shallow hole trap states is a stronger oxidizing agent than that with deep hole trap states because the shallow hole trap states at the lower energy level is a better electron acceptor.^73−75^ Therefore, the oxidation of OH to oxidation of O is enhanced on S2 compared to S1. Moreover, the triplet Ni[5] in S2 has a higher oxidation state (+4) than the doublet Ni[5] (+3) in S1, which also suggests that *OH with S2 is a stronger oxidizing agent (see DDEC charge analysis in Figure S12). These results implied that the dehydrogenation of *OH on NiAl(100) may follow the “surface–OH oxidation mechanism” where *OH is oxidized by the holes located at Ni[5] and O. When the external potential is lower than 0.39 V/SHE, the ground state of *OH/NiFe (S4) has both shallow and deep hole trap states composed of Ni[5] and O, respectively (Figure S11). Even though the oxidation state of Ni[5] in *OH/NiFe is +4, the same as that in *OH/NiAl, the O of the hydroxyl in *OH/NiFe carries nearly zero charge (−0.01 |e^–^|), which is more positively charged than the O (−1.0 |e^–^|) in *OH/NiAl. Therefore, NiFe is expected to demonstrate enhanced OER activity, potentially owing to the more potent oxidative nature of the radical form of oxygen (Ni^4+^–O·) compared to the ionic form of oxygen (Ni^4+^–O^–^) in NiAl.^71,75^

### *O

The potential of spin crossover is around 1.21 V/SHE between the ground state (S3) and the excited state (S2) for *O/NiAl, and it is around 1.45 V/SHE between S2 and S1 for *O/NiFe. The lower spin crossover potential for *O/NiAl is because the PZC of S3 of *O/NiAl (1.0 V/SHE) is lower than that of S2 of *O/NiFe (1.13 V/SHE) and the smaller capacitances of *O/NiFe (1.18 e/V), as shown in Table S8. A shallow hole trap state is located at the Fermi energy level in S3 of *O/NiAl, which enables the oxidation reaction, while the oxidation state of Ni[5] in S2 (“+4”) is higher than that in S3 (“+3”) as shown in the orbital diagram, which suggests S2 is a stronger oxizing agent than S3, leading to the subsequent nucleophilic attack of H_2_O. For *O/NiFe, both S2 and S1 have shallow and deep hole trap states, respectively; however, the shallow and deep hole trap states of S2 are higher energy than those of S1, suggesting stronger oxidation ability.

### *OOH

The spin crossover between S1 and S3 of *OOH occupies at much lower external potentials for both NiAl (0.2 V/SHE) and NiFe (0.1 V/SHE) compared to other intermediates. This is attributed to the lower PZC and the greater capacitance difference between S1 and S3 of *OOH. We observed a hollow hole trap state at the Fermi energy level of S3 and a deep hole trap state of S1 for both *OOH/NiAl and *OOH/NiAl, indicating that S3 is a stronger oxidizing agent than S1, which enhances the deprotonation of *OOH.

The spin crossover was observed for all of the intermediates for NiAl and NiFe systems. The potential at which the spin crossover occurs depends on the PZC, capacitance discrepancy, and energy difference at the PZC of the spin states. The increased oxidation state of Ni ions, along with the presence of a shallow hole trap state at the Ni ions, enhances the OER activity by strengthening the oxidation capability of the active Ni site.

### Effect of External Potential and pH on OER Activity

**Figure 4:**
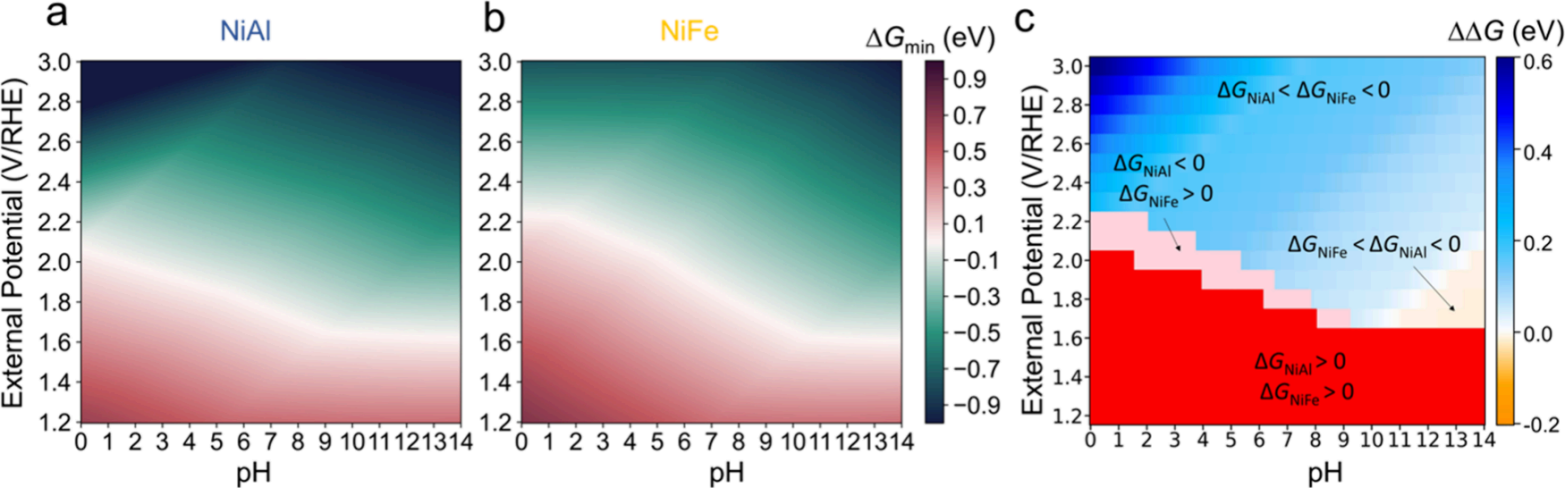
The minimum grand free energy of reaction (Δ*G*_min_) of the OER under varied pH and external potential for (a) NiAl(100) and (b) NiFe(100), and (c) the differences (ΔΔ*G* = Δ*G*_min(NiFe)_ – Δ*G*_min(NiAl)_) between Δ*G*_min_ for NiFe and NiAl as a function of pH and the external potential.

Based on the fitted quadratic function, the grand free energy of each intermediate can be calculated at any external potential and pH for all of the possible spin states. Figure 4a,b shows the mapping out of the minimum grand free energy of reaction (Δ*G*_min_) for the OER on NiAl(100) and NiFe(100) as a function of pH and the external potential with all the possible spin states considered. The reaction grand free energy for each elementary step and PDS for both surfaces is shown in Figure S13. For both catalysts, Δ*G*_min_ is greater than 0 eV in the red region while it is less than 0 eV in the green region. The red and green regions are separated by a white line, and the external potential values on the white line correspond to the onset potential (*U*_onset_) for the OER at different pH. For example, the onset potentials for the OER at pH 14 are 1.63 V/SHE for both NiAl and NiFe. The onset potential decreases with the increase of pH, aligning with the superior OER performance observed in basic media when compared to acidic conditions.^22,28,76^ However, the impacts of pH on the performance of NiAl and NiFe are different. For NiAl(100), *U*_onset_ decreases linearly with a slope of 0.05 V/pH as pH increases from 0 to 9 and remains relatively constant when pH > 9. On the other hand, for NiFe(100), *U*_onset_ shows little changes as pH increases from 0 to 2, which decreases at a greater rate with a slope of 0.075 V/pH as pH increases from 2 to 10. The effect of pH on the onset potential is described by the Nernst Equation. In the case of a single-electron transfer process, the electrode potential varies with pH at a rate of 0.0592 V/pH (see eq 4). NiAl exhibits a slope of 0.05 V/pH, closely aligned with the rate for the one-electron transfer process. However, NiFe demonstrates a steeper slope of 0.075 V/pH, surpassing the typical value of 0.0592 V/pH. This discrepancy suggests a higher number of electron transfers on NiFe compared to NiAl, resulting in a larger charge transfer coefficient and a smaller Tafel slope, thereby indicating higher activity of NiFe (see Section 16 of the SI). Since *U*_onset_ remains almost constant when the pH is greater than 9 for both catalysts, it is not necessary to increase the pH to a very high value in the experiments. Additionally, higher pH levels can reduce the proton transfer rate between the anode and cathode. Therefore, it is of importance to identify an appropriate pH to balance the thermodynamic and kinetic properties.^73^Figure 4c illustrates the relative energy difference (ΔΔ*G*) between Δ*G*_min_ for NiAl and NiFe. The Δ*G*_min_ values for NiAl and NiFe are positive in the red region, indicating that the OER will not occur when the external potential is lower than 1.63 V/RHE at a pH of 0–14. In the pink region, Δ*G*_min_ is less than 0 eV for NiAl while it is greater than 0 eV for NiFe, suggesting that the OER can occur on NiAl but failed on NiFe in the acidic media. In the blue and yellow regions, both NiAl and NiFe exhibit reactivity for the OER because the Δ*G*_min_ values for both catalysts are negative. However, their OER performance differs under varying reaction conditions. In the blue region, where the external potential is high and pH ranges from 1 to 14, NiAl exhibits superior activity compared to NiFe. However, under typical OER reaction conditions, characterized by lower external potentials and in the basic media, NiFe exhibits better activity than NiAl, as depicted in the yellow region.

### Practical Evaluation of Electrocatalytic Performance

**Figure 5:**
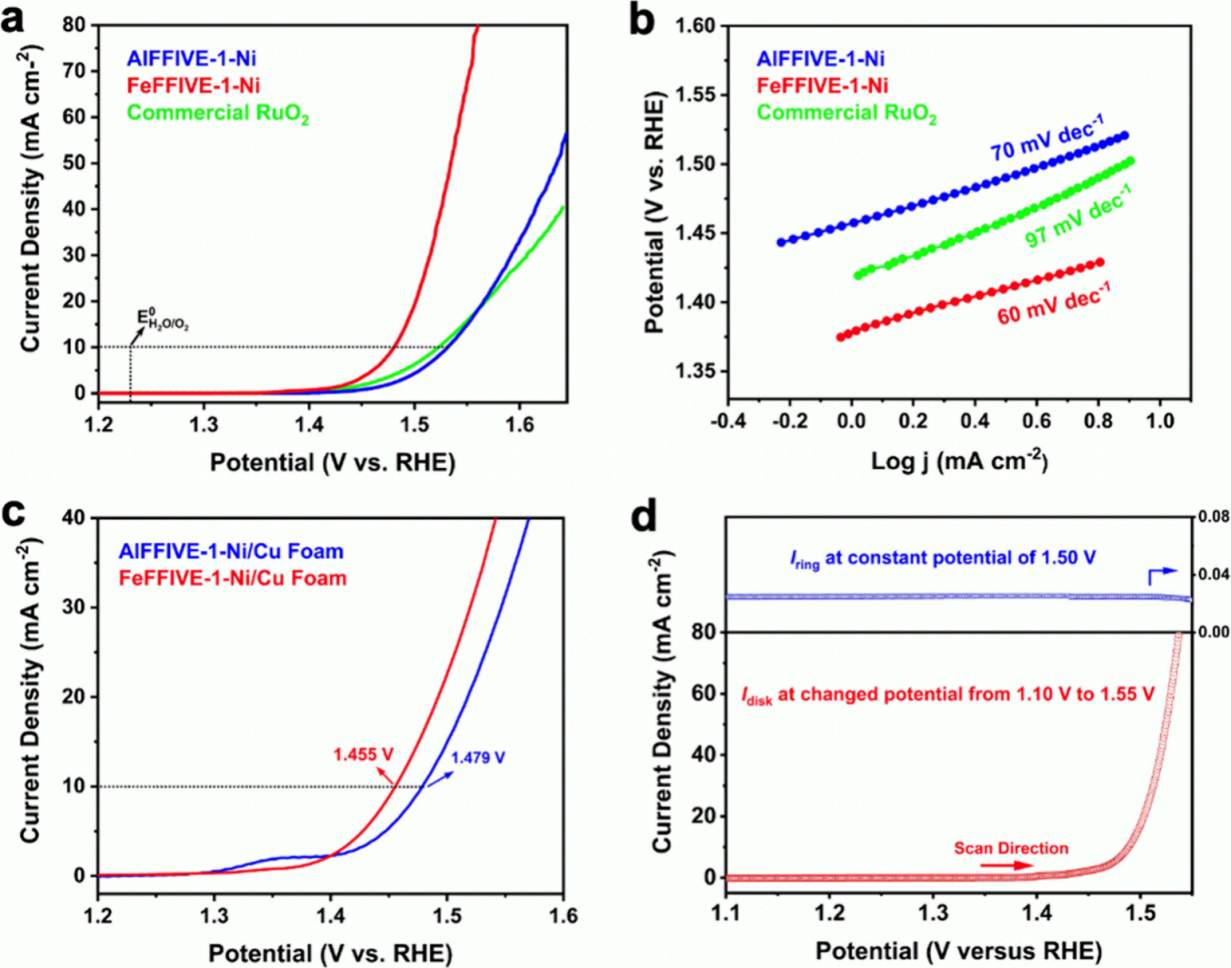
Electrocatalytic OER performance of NiAl and NiFe BMOFs. (a) Polarization curves of NiAl, NiFe, and commercial RuO_2_ in a 1 M KOH electrolyte at a scan rate of 5 mV s^–1^. The dotted horizontal line is the guide for a current density of 10 mA cm^–2^. (b) Tafel plots obtained from the polarization curves of NiAl, NiFe, and commercial RuO_2_. (c) Polarization curves of NiAl and NiFe loaded on Cu foam in a 1 M KOH electrolyte. The dotted horizontal line is the guide for current density of 10 mA cm^–2^. (d) Rotating ring-disk electrode voltammogram of NiFe in a 1 M KOH electrolyte.

The electrocatalytic activity was assessed in a traditional three-electrode cell in a 1 M KOH aqueous solution (Figure S18). To evaluate the OER performance, electrodes were prepared by uniformly depositing the synthesized BMOFs and commercial RuO_2_ onto a glass-carbon supporting electrode. Linear sweep voltammetry was employed to obtain polarization curves of NiFe, NiAl, and commercial RuO_2_ electrodes. In these polarization curves, a pronounced increase in anodic current response commenced at an onset potential (*E*_onset_) of 1.36 V/SHE (defined as the potential required to achieve a current density of 0.1 mA cm^–2^) from the NiFe electrode, slightly lower than the 1.41 V/SHE of the NiAl electrode (Table S12). The crucial parameter for the OER performance evaluation, the overpotential at 10 mA cm^–2^, highlighted the superior electrocatalytic activity of the NiFe electrode, exhibiting the lowest overpotential of 253 mV. This value was notably lower than that of NiAl (303 mV) and even commercial RuO_2_ (293 mV) (Figure 5a). The catalytic kinetics were further analyzed by using Tafel plots, as illustrated in Figure 5b. Depositing NiFe and NiAl BMOFs onto conductive copper foam resulted in improved OER performance, displaying overpotentials of 226 and 250 mV at 10 mA cm^–2^, respectively (Figure 5c). Notably, the measured Tafel slope of NiFe (60 mV dec^–1^) was significantly smaller compared to those of NiAl (70 mV dec^–1^) and commercial RuO_2_ (97 mV dec^–1^), signifying superior reaction kinetics. Moreover, experiments employing a rotating ring-disk electrode demonstrated that the product catalyzed by NiFe was exclusively O_2_ (Figure 5d). The ring and disk current were recorded while varying the disk potential from 1.1 to 1.55 V. A negligible current density attributed to the oxidation of hydrogen peroxide was observed on the ring electrode, confirming the desirable four-electron process of water oxidation: 4OH^–^ → O_2_ + 2H_2_O + 4e^–^. Beyond electrocatalytic activity, the operating stability and durability of the OER electrocatalyst are also crucial for potential large-scale applications. To characterize the stability of the NiFe catalyst, multistep potential statical cycling experiments between 1.49 and 1.51 V were conducted for ∼3000 s, demonstrating excellent OER recoverability (Figure S19). Furthermore, continuous electrolysis at a constant overpotential of 253 mV showed no significant decrease in current density over 4 h, outperforming commercial RuO_2_ (Figure S20). The synthesized NiAl and NiFe BMOFs show high crystallinity and purity, as shown in the XRD, SEM, and TEM results (Figures S21–S25). The oxidation states of Ni, Fe, and Al are 2+, 3+, and 3+, respectively, confirmed by XPS results (Figures S26 and S27). The stability of BMOFs is a significant concern for OER, such as causing by the oxidation of BMOFs to their oxides or collapse of the MOFs. We are currently investigating the mechanism for catalyst degradation and developing new strategies to improve their stability.

Our computational predictions are consistent with experimental findings in which NiFe demonstrates superior OER activity compared with NiAl under standard OER conditions. However, the polarization curves reveal that the overpotential for NiFe is consistently lower than that of NiAl, not only at low external potentials but also at high potentials at a pH of 14. This observation contradicts our computational predictions for high external potentials. To reconcile this discrepancy, we investigated the transition state in the presence of water as a solvent and examined the electrical conductivity of both BMOFs.

### Transition State

**Figure 6:**
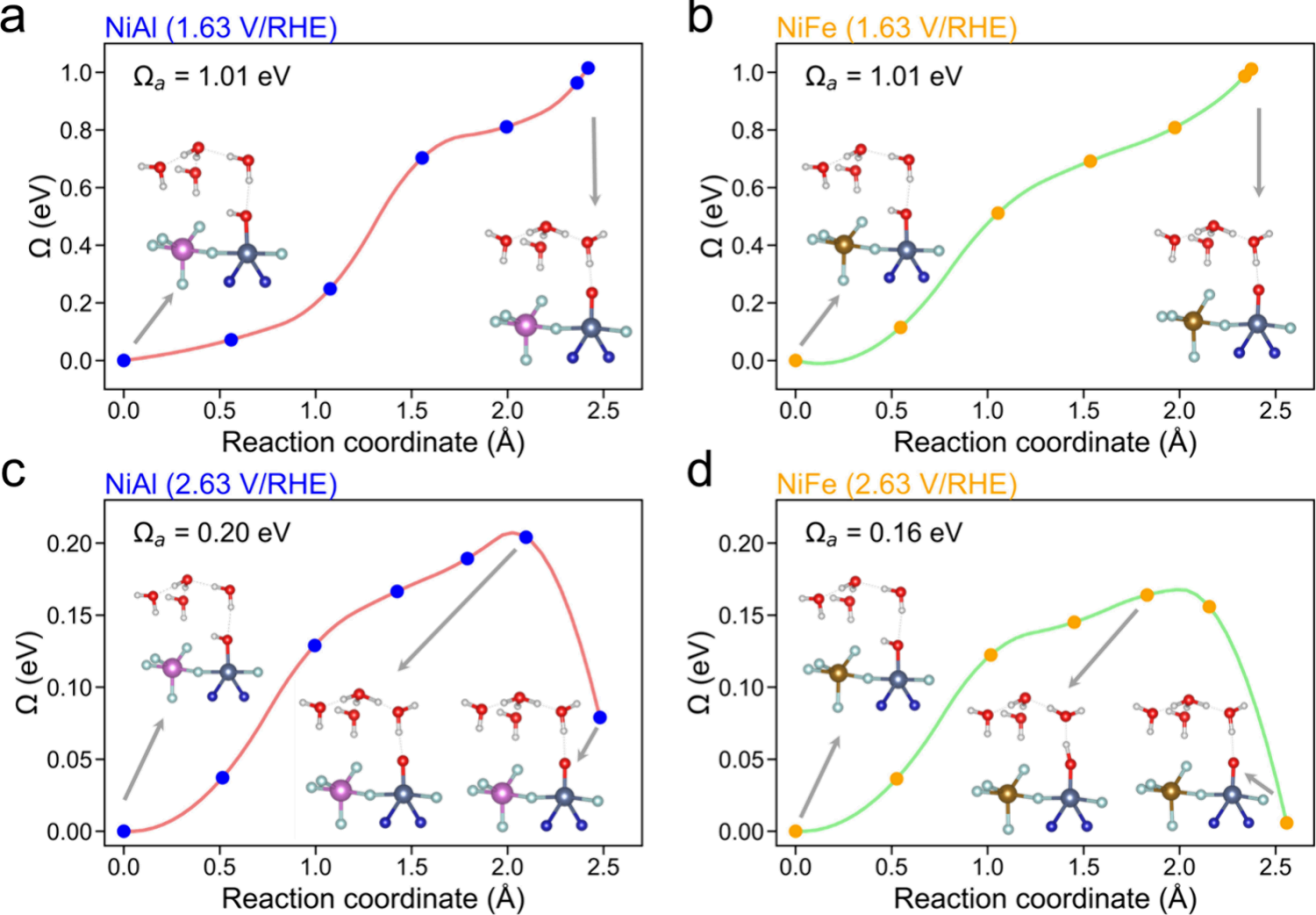
Free energy profiles for the potential-determining step (*OH →*O) on NiAl(100) and NiFe(100) at 1.63 V/RHE and 2.63 V/RHE at pH = 14.

Previous calculations did not account for the explicit solvent effect, and only the free energies were computed by assuming that activation energies are the same as the reaction energy. This approximation is true at a lower external potential even with the explicit solvent effect included. As demonstrated in Figure 6a and b, the potential-dependent transition states of PDS (*OH → *O) were not observed at 1.63 V/RHE for both NiAl(100) and NiFe(100), respectively. However, transition states were observed at the higher external potential of 2.63 V/RHE, and the activation free energies are 0.21 eV for NiAl and 0.16 eV for NiFe. The result suggests that one can no longer approximate the activation free energies equal to the reaction energy at the high external potential. At 2.63 V/RHE, NiFe(100) obtains a lower activation energy and a lower reaction energy than that of NiAl(100), suggesting a higher activity of NiFe at higher external potential aligning with the results from polarization curves. Moreover, the computed the relative Tafel slope (1.20) from the transfer coefficients based on PDS agrees well with the experimental value of 1.17 (see Section 16 of the SI).

### Electrical Conductivity

**Figure 7:**
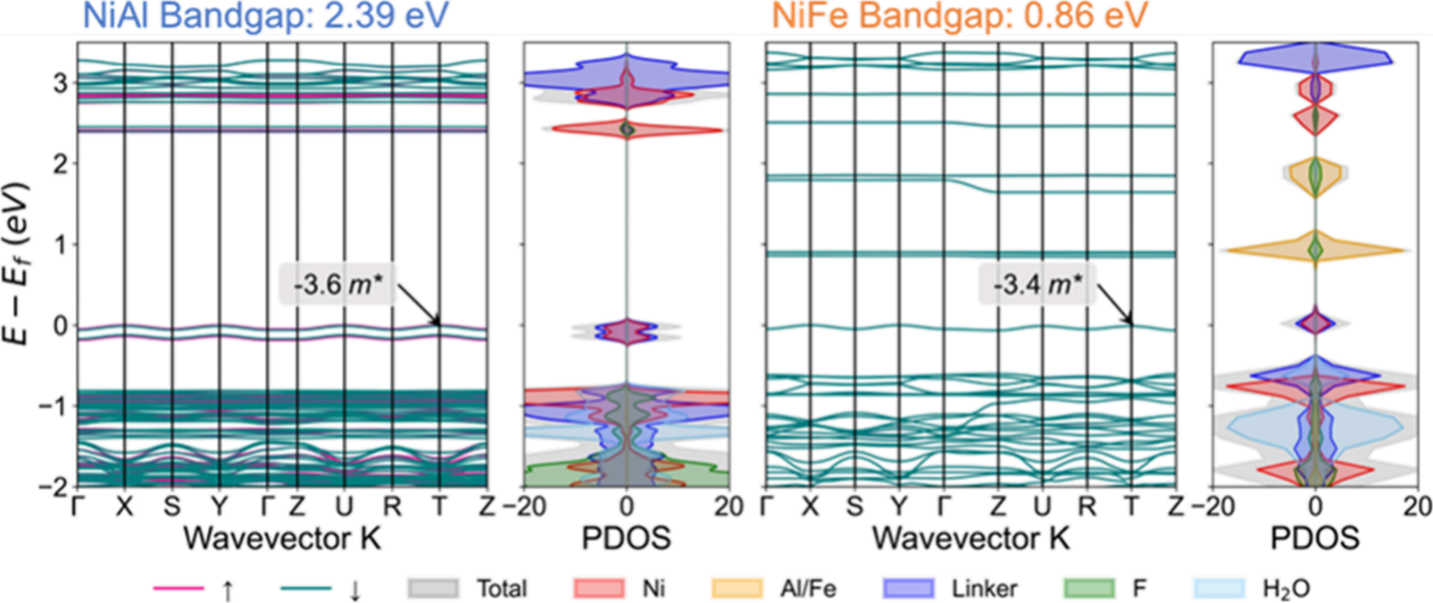
Band structures and projected density of states for bulk NiAl and NiFe.

The electrical conductivity of catalysts plays a significant role in the kinetics of electrocatalysis because it directly affects the charge transport between the electrode and the reactants in the electrolyte, ultimately affecting the reaction rate and the effective potential due to the voltage drop from the high resistance of electrocatalysts.^77^ Hence, we investigated the conductivity of NiAl and NiFe bulk materials by analyzing the energy band structure, PDOS, and effective mass. As shown in Figure 7, NiFe exhibits a narrower band gap (0.86 eV) compared to NiAl (2.39 eV), primarily attributed to the introduction of new bands around 1 eV resulting from the substitution of Al with Fe. Therefore, the conduction band minimum (CBM) of NiFe is significantly lower compared to that of NiAl. Furthermore, calculated curvature hole effective masses in the parabolic approximation of NiFe are smaller than those of NiAl (see Table S10). The narrower band gap and the smaller effective mass of holes suggest that NiFe has a higher conductivity compared to NiAl. We did not include the effective mass of electron in this discussion since the CBM is almost flat for both NiAl and NiFe. From the PDOS results, we observed that the main contributors to the valence band maximum (VBM) of both materials are Ni and the linkers, which overlap significantly, facilitating the efficient charge transfer between Ni and the linkers. However, the CBM of NiAl comprises Ni and F, which is different from NiFe that is composed of Fe and F. As depicted in Figure S14a,d, the spin-up density of VBM and the spin-down density of CBM locate at the same Ni ion for NiAl, which facilitate the recombination of the electrons and holes.^78^ In contrast, for NiFe, both spin-up and spin-down densities are located at different Ni and Fe ions (Figure S15a–d), indicating a more effective separation of holes and electrons in NiFe. As a result, the concentrations of electrons or holes are expected to be higher in NiFe than those in NiAl owing to the slower electron–hole recombination, leading to a better conductivity and better OER performance on NiFe.

### Exchange Effect Promoted Electrical Conductivity

It is known that electron from one metal ion (M_A_) can hop to the next-to-nearest neighboring metal ion (M_B_) with different d orbital occupancies through an intermediate anion or ligands, X (M_A_–X–M_B_), leading to conductive pathways through the super-exchange (SE) or double-exchange (DE) interaction. When the spins of two neighboring metal ions are antiparallel, one electron hops from M_A_ to M_B_ via X due to SE interaction, driven by partially filled d orbitals of M_B_. Conversely, when the spins of two neighboring metal ions are parallel, one electron hops from M_A_ to M_B_ via a DE interaction, triggered by the unoccupied d orbitals of M_B_. Both SE and DE interactions were found in perovskite and high valency transition metal oxides, which improve the OER performance by enhancing the electrical conductivity.^14,79−83^

**Figure 8:**
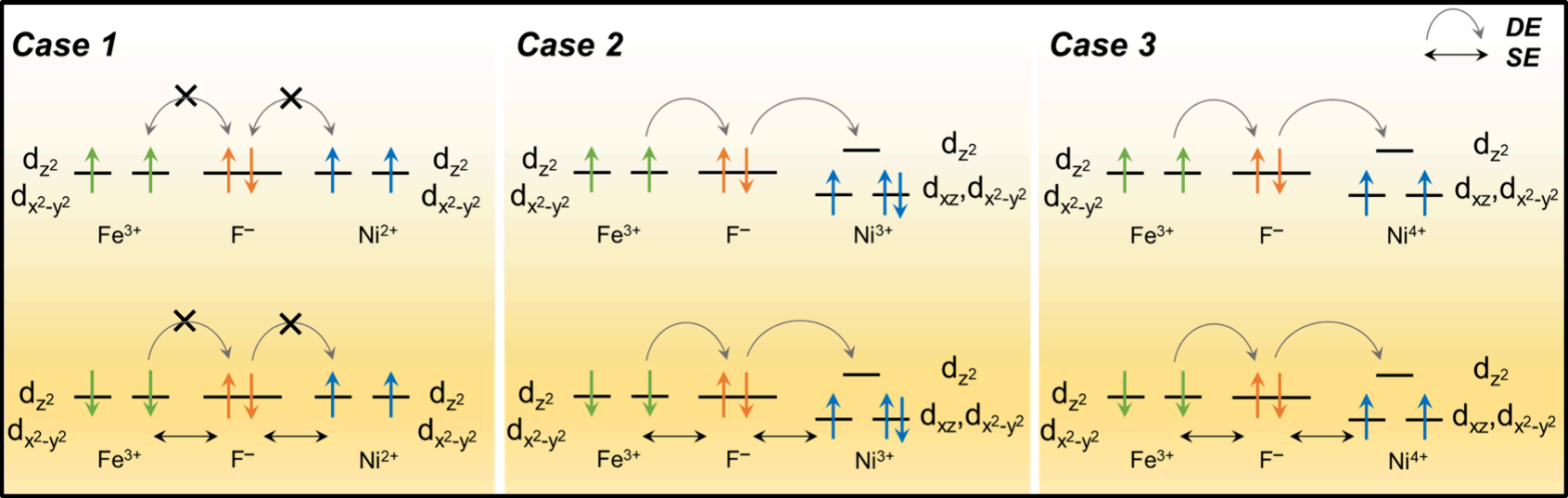
Super-exchange and double-exchange mechanisms along the Fe–F–Ni chain. d_*xy*_, d*_xz_,* and d_*yz*_ orbitals are singly occupied for Fe^3+^, while they are doubly occupied for Ni^2+^; d_*xy*_ and d_*yz*_ orbitals of Ni^3+^ and Ni^4+^ are doubly occupied. The diagrams for these orbitals are not included for the sake of clarity.

NiAl does not have SE or DE because the orbitals of Al^3+^ are fully occupied. However, NiFe possesses strong SE and DE interactions, as shown in Figure 8. For the NiFe bulk material and surface, Ni ions of NiFe hold an oxidation state of “+2”, as shown in case 1. When the electron spins of Ni^2+^ line up parallel to those of Fe^3+^, there is no exchange interaction, while when their electron spins are antiparallel, SE is triggered by the singly occupied Ni 3d orbitals. During the reaction, the oxidation state and spin state of Ni ion changes are induced by the external potential, the adsorption of an adsorbate, and pH. As shown in cases 2 and 3, when the electron spins of Ni^3+^/Ni ^4+^ and Fe^3+^ are parallel, electrons of Fe^3+^ can hop to the empty d_z2_ of Ni^3+/4+^ due to the DE interaction, while when they are antiparallel, the electrons of Fe^3+^ can hop to half-filled d_*xz*_ or d_x2–y2_ of Ni^3+/4+^ due to SE. Therefore, an SE and DE interaction might coexist in NiFe when Ni ions have a high oxidation state. Combined with previous PDOS analysis, we conclude that a high oxidation state of Ni can not only lead to a shallow hole trap state but also result in SE and DE interactions, which can promote the OER performance by increasing the conductivity.

### Future Investigation Guidance

In our study, we observed spin crossover induced by alternating the external potential and spin flip in the AIMD simulation (Figure S2). In addition, light and magnetic fields have been reported to induce spin excitation and flips.^84−87^ Therefore, understanding the external stimuli including external potential, light, and magnetic field on the OER performance in combination of theory and experiment are emerging research directions. As illustrated in Figure S16, with all intermediates at their ground state, *U*_onset_ is 1.63 V/RHE for both surfaces. However, when *OH is excited to S1, where Ni[5] transitions from triplet to doublet, *U*_onset_ decreases to 1.43 V/RHE at pH = 14. Furthermore, by inducing a spin flip in Ni[2], transitioning it from a triplet state to a singlet state, the same *U*_onset_ can be achieved at a slightly lower pH of 12. The nonadiabatic molecular dynamics (NAMD) approach developed by Zhao et al. to study real-time charge carrier quantum dynamics in momentum space could serve as an effective tool for exploring the dynamics of spin changes under external field conditions.^88^ Furthermore, canonical NVTΦ ensemble might be a promising approach to handle external potentials during the dynamic process.^89,90^ The effect of pH on the potential in this work is described through the Nernst equation. Moreover, the fluctuation in pH can affect the catalytic process by altering the ion concentration in the electrical double layer, offering an intriguing area for future investigation.

Super-exchange and double-exchange interactions were found to coexist in the NiFe MOF, enhancing the electrical conductivity and reaction kinetics. Goddard et al. also demonstrated that doping γ-NiOOH with elements like Fe, Co, Rh, or Ir can optimize OER thermodynamics due to DE interaction.^14^ The superexchange interactions are closely connected to the symmetry of electron orbitals. Therefore, computationally screening different combinations of M_A_–X–M_B_ and different coordination geometries that offers better SE and DE interactions would potentially promote OER performance.

We observed that Fe–F bonding falls into antibonding regions around the top of valence bands of Ni, Fe, and F ions at the surface according to results of COHP (Figure S17 and Table S11). The electrons in the antibonding orbitals have stronger mobility than those in the bonding orbital. Therefore, the COHP results also indicate a stronger exchange interaction in NiFe than NiAl because the electrons on Ni–F antibonding orbitals are more likely to migrate to nearby Fe ions. The bonding analysis via COHP was employed to identify the exchange interaction qualitatively.

## 4 Conclusions

We employed density functional theory to investigate the OER activities of NiAl and NiFe BMOFs under a wide range of external potentials and pH levels. Specifically, we calculated all the possible spin states for each intermediate (*, *OH, *O, and *OOH) at PZC and screened the energy variations of each spin state as a function of the external potential. We observed that the spin state featuring a shallow hole trap state around the Fermi energy level and the higher oxidation state of Ni ions serve as strong oxidizing agents, promoting the OER. The spin crossover induced by the external potential were observed for each intermediate, leading to significant changes in overall reaction and activation energies due to altered energy levels. Combining the constant potential method and electrochemical nudged elastic band method, we mapped the minimum free energy barriers of the OER under varied external potential and pH by considering the spin crossover effect, implicit coupled with explicit solvation effect for both NiAl and NiFe BMOFs. We found that NiFe exhibits better OER thermodynamics and kinetics than NiAl, which is in good agreement with experimental measured OER polarization curves and Tafel plots. Moreover, we found that the enhanced OER kinetics of NiFe is not solely attributed to lower barriers but is also a result of improved electrical conductivity arising from the synergistic effects of dual Ni–Fe metal sites. Specifically, replacing the second metal Al with Fe (i) reduces the band gap and the effective mass of holes compared to NiAl BMOF and (ii) initiates super- and double-exchange interactions within the Ni–F–Fe chain, thereby enhancing electron transfer and hopping, ultimately leading to superior OER kinetics. Our study unveils the synergistic impact of dual-metal catalysts on the OER activity, encompassing spin crossover, super and double exchange, band gap, and band structure. Additionally, we shed light on how reaction conditions, such as external potential and pH, influence the OER activity. These findings deepen our understanding of the mechanism of the OER and offer guidance for developing efficient bimetallic catalysts in electrocatalysis.

## Supporting Information Available

Energy data (XLSX)
NiAl and NiFe BMOF unit cells, and all the reaction intermediates (ZIP)
DFT-computed structures, electronic energies, magnetic moments, and electronic structure analysis for unit cell, surfaces, and intermediates; experimental details, materials, and methods and characterization, including NiAl and NiFe MOF synthesis, OER experimental setup, OER performance test, TEM, SEM, PXRD, XPS, and N_2_ adsorption–desorption isotherms (PDF)
