# Supplementary material for: Spin Crossover and Exchange Effects on Oxygen Evolution Reaction Catalyzed by Bimetallic Metal Organic Frameworks
Source: ACS Catal. 2024 May 20;14(11):8652–65. doi: 10.1021/acscatal.4c01091 (PMC11165450; doi:10.1021/acscatal.4c01091)
Supplement: Supplementary file 3 — cs4c01091_si_003.pdf [file cs4c01091_si_003.pdf]

Supporting Information

The Spin Crossover and Exchange Effects on Oxygen Evolution Reaction Catalyzed by Bimetallic Metal Organic Frameworks

Guangsheng Liu,[a,†] Feng Xie,[b,†] Xu Cai,[c] Jingyun Ye[a,*]

[a] Department of Chemistry and Biochemistry, Duquesne University, Pittsburgh, PA 15282, USA.
[b] Department of Chemistry and Chemical Biology, Rutgers University, Piscataway, NJ 08854, USA.
[c] State Key Laboratory of Photocatalysis on Energy and Environment, College of Chemistry, Fuzhou University, Fuzhou 350108, PR China.

* Correspondence to: jye1@duq.edu (J. Y.)
† Co-first author

Table of Contents

1. Functional test for NiAl unit cell optimization S2
2. Surface model selection S2
3. The stability and magnetic moment fluctuation of NiAl(100) S3
4. K points test for NiAl(100) surface. S4
5. U value test S5
6. Gibbs free energy calculation S6
7. Comparison of the Gibbs free energy diagrams from the different functional........ S7
8. Flow chart for the configuration identification........ S9
9. Oxidation states assignment via magnetic moment........ S9
10. Relative energy and magnetic moment for selected spin state S10
11. Electronic and geometric structure analysis S12
12. Spin-crossover under external potential S15
13. Spin density and DDEC charge S16
14. Reaction grand free energy and PDS S17
15. Fitted parameters of the quadratic equation........ S17
16. Computing the proportion of transfer coefficient S18
17. Effective mass at different K points. S19
18. Squared wavefunction analysis for NiAl and NiFe bulk materials S20
19. Future Investigation Guidance........ S21
20. Experimental Detail S23
21. References........ S23

1. Functional test for NiAl unit cell optimization

Table S1. Lattice constants and bulk volumes from the experimental and computational results, where the U of Ni is 4.89.

		a/Å	b/Å	c/Å	Volume/Å^3
	Experimental	9.939	9.939	15.414	1522.500
PBE	Theoretical	9.914	9.807	15.680	1524.471
	Relative error[a]	-0.002	-0.013	0.017	0.001
PBE+U	Theoretical	9.858	9.736	15.536	1490.926
	Relative error	-0.008	-0.020	0.008	-0.021
PBEsol+U	Theoretical	9.651	9.565	15.282	1410.520
	Relative error	-0.029	-0.038	-0.009	-0.074
PBEsol	Theoretical	9.763	9.613	15.537	1458.035
	Relative error	-0.018	-0.033	0.008	-0.042

[a]The relative error is equal to $\frac{Theoretical - Experimental}{Experimental}$

2. Surface model selection

The crystal structure and morphology of AlFFIVE-1-Ni has been investigated extensively by Datta et al.[1] As illustrated in **Figure S1**, the Ni-site on NiAl(100) has a coordination number of 4, while which has a coordination number of 5 on both (110) and (001) surfaces. Therefore, we have selected the (100) model to investigate the oxygen evolution reaction (OER) occurring on NiAl, because a metal site with lower coordination number, namely more unsaturated sites, tends to show a higher activity.

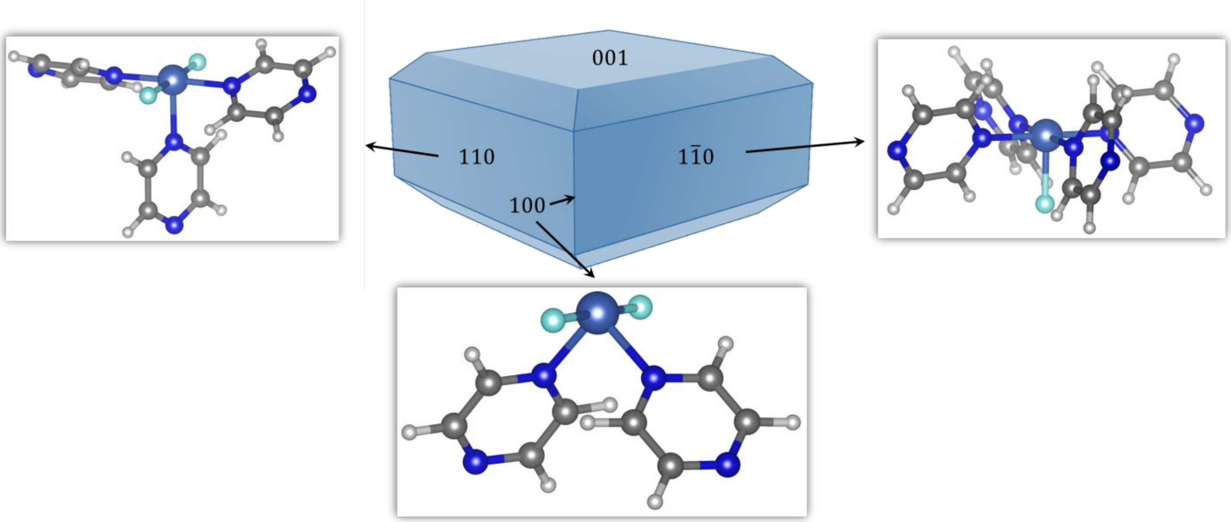

Figure S1. The Schematic illustration of KAUST-8 crystal.

3. The stability and magnetic moment fluctuation of NiAl(100)

The NiAl(100) structure demonstrated stability during the 10 ps AIMD simulation under the NVT ensemble, despite noticeable fluctuations in magnetic moment. As depicted in **Figure S2a**, throughout the AIMD simulation, neither the average nor the range of absolute magnetic moments for the 6 Ni ions remained constant. Instead, both the avg_mag and range_mag exhibited changes corresponding to shifts in energy and temperature variations. Notably, the range_mag varied between 0 μ_B to 1.5 μ_B, indicating the presence of a singlet Ni resulting from a spin flip. Similar demagnetization processes have been observed in metallic nickel films when exposed to femtosecond optical pulses.[2] Hence, it's plausible to hypothesize that such a phenomenon could also manifest during the OER process, potentially impacting OER activity.

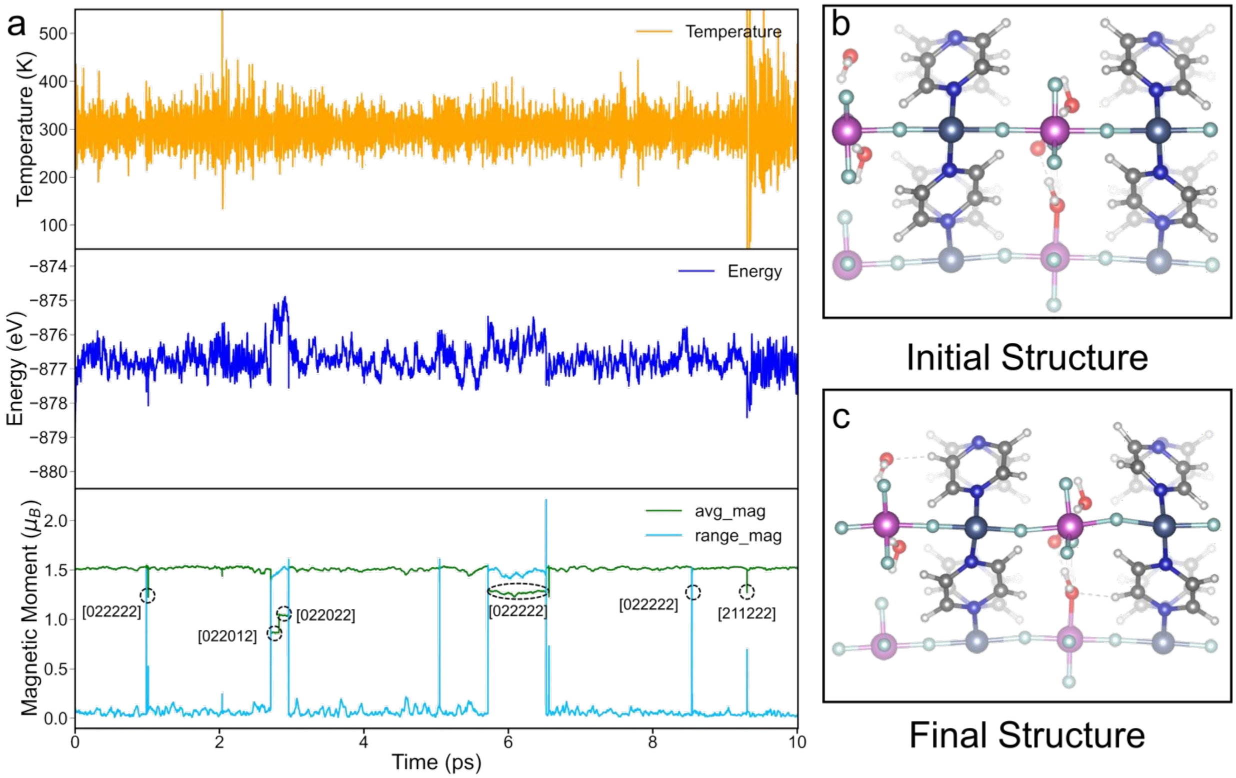

Figure S2. Ab initio molecular dynamics simulation for NiAl(100). (a) Plots showcasing the relationship between temperature, energy, magnetic moment, and time; here, 'avg_mag' represents the average of absolute magnetic moments of 6 Ni ions , while 'range_mag' denotes their range. (b) Initial structure captured at 0 ps. (c) Final structure observed at 10 ps.

4. K points test for NiAl(100) surface.

We have tested the effect of K point on the energies. **Figure S3** shows the free energy profiles for OER catalyzed on NiAl(100) calculated with 1×1×1 Γ k-point grid and 3×2×1 k-point grid. The onset potential of OER is very similar based on this test: 0.81 V (1×1×1 Γ k-point) vs 0.84 V (3×2×1 k-point). Therefore, the 1*1*1 Γ k-point grid were used for all the calculations to balance the computational cost.

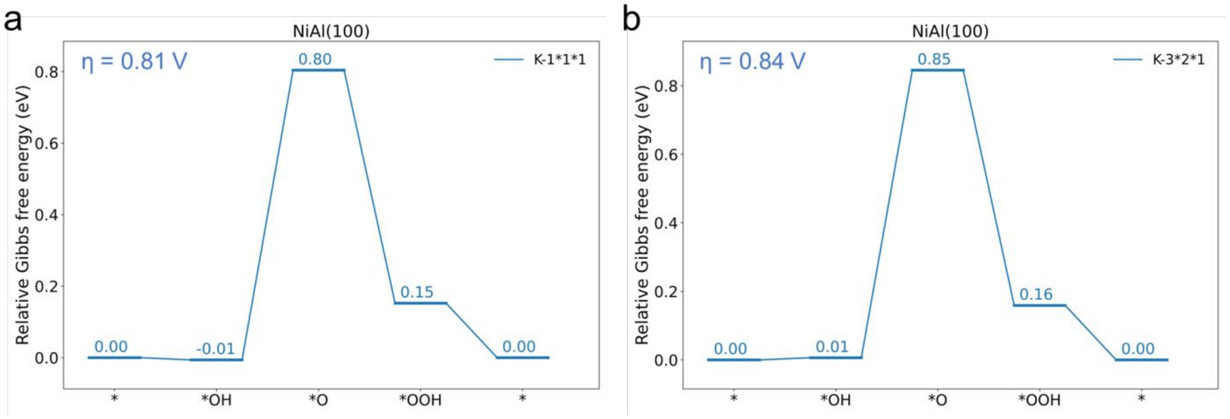

Figure S3. K points test for NiAl(100) surface: **(a)** 1*1*1 and **(b)** 3*2*1.

5. U value test

We calculated the U values of Ni with and without adsorbates using the linear response method to evaluate the accuracy of PBE+U and compare with HSE functional. As shown in **Figure S4**, Ni with different adsorbed specie have different value of U (see **Figure 4a**, **4c**, **4e**). Even though the Ni in same place without adsorbate still displayed different U values if the adjacent Ni with different adsorbates (see **Figure 4b** and **4d**). Therefore, we use HSE functional with different mixing factors (α = 0.15 and 0.25), rather than PBE+U method.

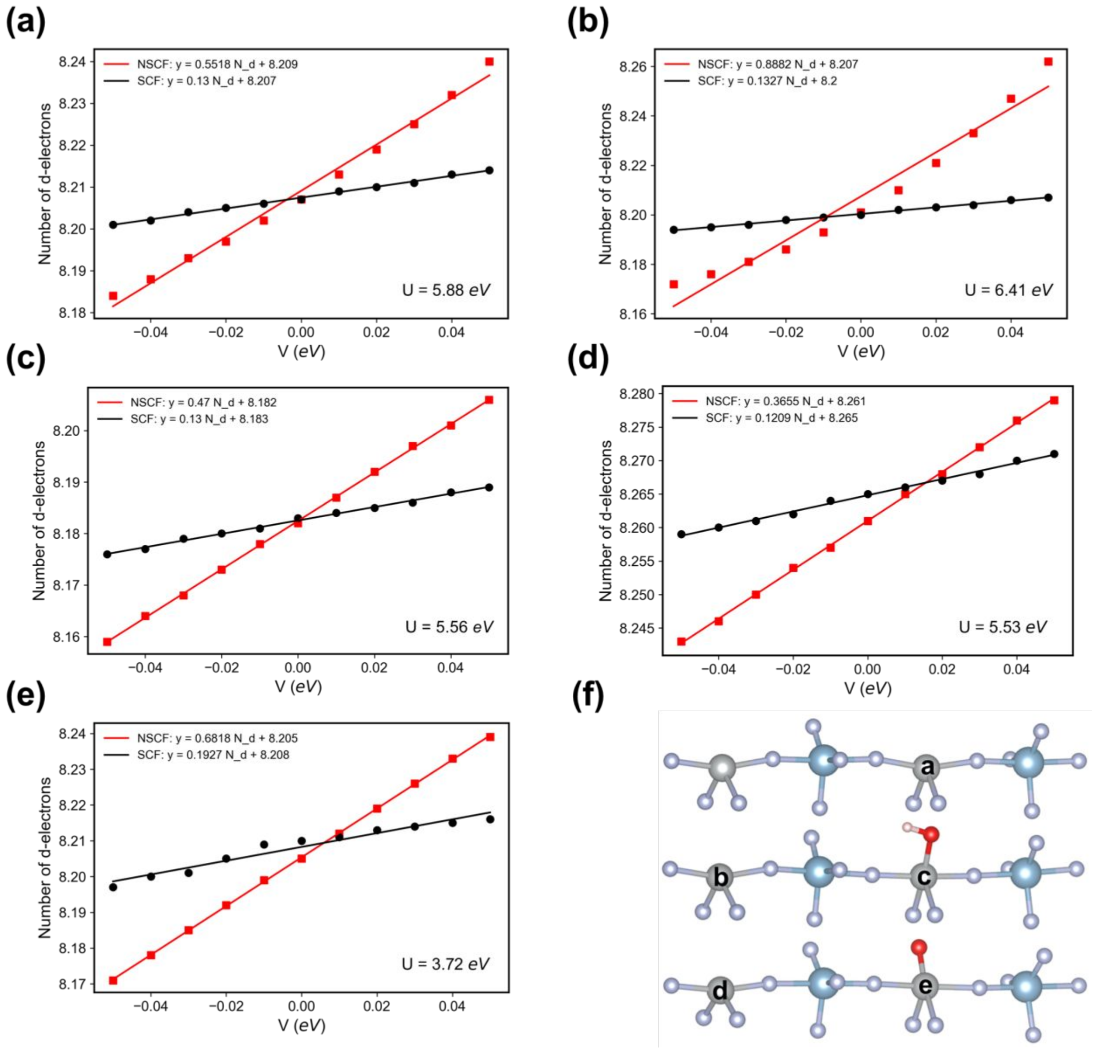

Figure S4. **(a-e)** U value for Ni ion at the top surface under different chemical environment (with *O or *OH adsorbate, or without adsorbate), and the location of Ni ion of **(a)** to **(e)** tests are labeled in **(f)**.

6. Gibbs free energy calculation

Constant Charge Method Proposed by Nørskov: In the scenario of this method, OER could occur in the following four PCET elementary steps, in which the process is pH-independent since the referential electrode has the same pH as work electrode.

$$H_2O(l) + * \rightarrow HO^* + H^+ + e^- \# (S1)$$
$$HO^* \rightarrow O^* + H^+ + e^- \#(S2)$$
$$H_2O(l) + O^* \rightarrow HOO^* + H^+ + e^- \#(S3)$$
$$HOO^* \rightarrow O_2(g) + H^+ + e^- \#(S4)$$

Above * denotes the active site on the surface of electrocatalysis. Due to apply the computational hydrogen electrode (CHE) model, the Gibbs energy of (H^+ + e^-) is referenced as 1/2G(H_2). The change of the Gibbs energy for the reaction path following Equations S1 to S4 can be expressed as Equation S5 to S8 respectively,

$$\Delta G_1 = E_{DFT}^{HO^*} + \frac{1}{2}E_{DFT}^{H_2(g)} - E_{DFT}^{*} - G_{DFT}^{H_2O(l)} + (\Delta ZPE - T\Delta S_{ads}) - eU\#(S5)$$
$$\Delta G_2 = E_{DFT}^{O^*} + \frac{1}{2}E_{DFT}^{H_2(g)} - E_{DFT}^{HO^*} + (\Delta ZPE - T\Delta S_{ads}) - eU\#(S6)$$
$$\Delta G_3 = E_{DFT}^{HOO^*} + \frac{1}{2}E_{DFT}^{H_2(g)} - E_{DFT}^{O^*} - G_{DFT}^{H_2O(l)} + (\Delta ZPE - T\Delta S_{ads}) - eU\#(S7)$$
$$\Delta G_4 = G_{non-DFT}^{O_2(g)} + \frac{1}{2}E_{DFT}^{H_2(g)} - E_{DFT}^{HOO^*} + (\Delta ZPE - T\Delta S_{ads}) - eU\#(S8)$$

where E_{DFT}^{*}, $E_{DFT}^{HO^*}$, $E_{DFT}^{O^*}$, $E_{DFT}^{HOO^*}$, $E_{DFT}^{H_2(g)}$, and U represent the DFT total energy of clean surface slab, HO adsorbed surface slab, O adsorbed surface slab, HOO adsorbed slab, H_2 molecule, and electrode potential, respectively. Noted that PBE would overestimate the binding energy of O_2(g), hence we calculate the free energy of O_2(g) from the calculated free energies of H_2(g) and H_2O(l) from the equations (S9) and (S10), in which the entropy for H_2O is calculated at 0.035 bar since this is the equilibrium pressure of H_2O at 298 K,[3] together with the experimental standard free energy of formation of H_2O(l) at 298 K:[4]

$$G_{DFT}^{H_2(g)} = E_{DFT}^{H_2(g)} + ZPE_{DFT}^{H_2(g)} - T\Delta S_{298\,K}\#(S9)$$
$$G_{DFT}^{H_2O(l)} = E_{DFT}^{H_2O(g)} + ZPE_{DFT}^{H_2O(g)} - T\Delta S_{298\,K}^{0.035\,bar}\#(S10)$$
$$G_{non-DFT}^{O_2(g)} = 4.92\ eV + \left(2G_{DFT}^{H_2O(l)} - 2G_{DFT}^{H_2(g)}\right)\#(S11)$$

The same expression is used for HSE06 for consistency, although the errors in H_2O formation energy ($\Delta E^f_{H_2O(g)}$) are smaller than PBE. Note that the standard reduction potential for O_2 ($E^{\circ}_{O_2/H_2O}$) is 1.23 eV independent of the XC functional. [5–8]

Gas Molecule and Adsorbed Molecule Free Energy Correction:

In this work, all the free energy correction processed by vaspkit,[9] since the VASP doesn't supply a module to calculate the free energy directly. As mentioned by the tutorial of vaspkit, the thermochemical data for gas molecules are computed following the same step of Gaussian Software,[10] while the adsorbed molecules are regarded as hindered rotor model, in which the translational and rotational contributions are attributed to the vibration part. Noted that the contribution of frequency below 50 cm^{-1} is calculated as 50 cm^{-1} for the adsorbed molecules, since a small vibration frequency will lead to abnormal entropy and free energy correction. The contribution of electron motion is also neglected as its small contribution. All the computing formulars can be found in the tutorial of vaspkit and Gaussian. All the thermochemical data are listed in the **Table S2**.

Table S2. Thermochemical data for gas and adsorbed molecules calculated by different functionals.

Functional	Species	E_{DFT} / eV	ZPE / eV	TS / eV	$G(T)_{corr}$ / eV	G / eV
PBE	H_2O	-14.22	0.57	0.67	0.00	-14.22
	H_2	-6.77	0.27	0.40	-0.04	-6.81
	O_2	--	--	--	--	-9.90
PBE Solvent	H_2O	-14.57	0.56	0.67	0.00	-14.57
	H_2	-6.79	0.27	0.40	-0.04	-6.83
	O_2	--	--	--	--	-10.56
HSE $\alpha = 0.15$	H_2O	-16.16	0.57	0.67	0.00	-16.16
	H_2	-7.41	0.27	0.40	-0.04	-7.45
	O_2	--	--	--	--	-12.51
HSE $\alpha = 0.25$	H_2O	-17.47	0.57	0.67	0.00	-17.47
	H_2	-7.83	0.27	0.40	-0.04	-7.88
	O_2	--	--	--	--	-14.26
All[a]	HO*	--	--	--	0.34	--
	O*	--	--	--	0.00	--
	HOO*	--	--	--	0.41	--

[a]The correction value of free energy are regarded as the same for all functionals.

7. Comparison of the Gibbs free energy diagrams from the different functional.

The energy diagram for NiAl(100) and NiFe(100) is presented, employing various functionals and reactive sites. The functionals in use include the PBE (Perdew-Burke-Ernzerhof)

and HSE (Heyd-Scuseria-Ernzerhof), with the latter incorporating a 25% Hartree-Fock exact exchange component.

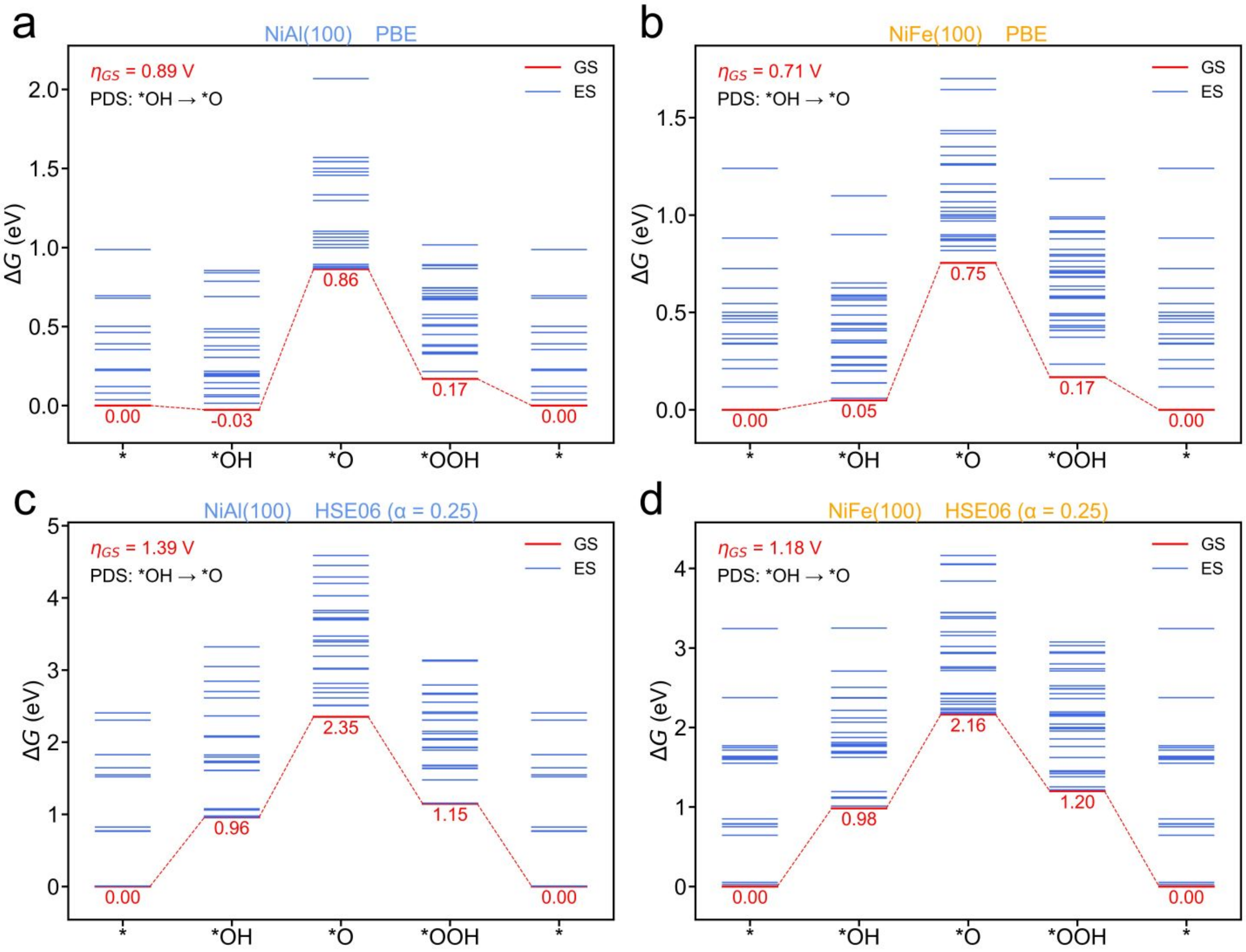

Figure S5. The Gibbs energy diagrams of OER at Ni-site of NiAl/NiFe (100) surface with different functionals. **(a)** PBE for NiAl(100) and **(b)** NiFe(100), **(c)** HSE06 with α = 0.25 for NiAl(100) and **(d)** NiFe(100).

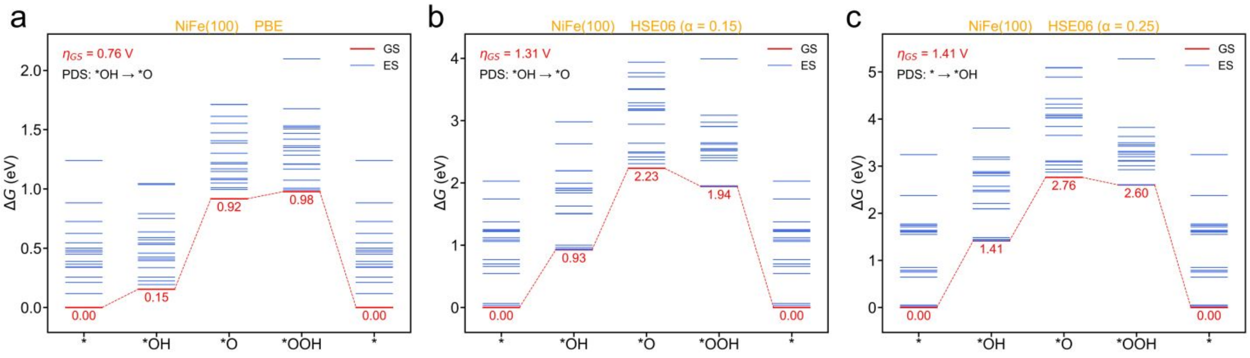

Figure S6. The Gibbs energy diagrams of OER at Fe-site of NiFe(100) with different functionals: **(a)** PBE, **(b)** HSE06 with α = 0.15 and **(c)** HSE06 with α = 0.25.

8. Flow chart for the configuration identification

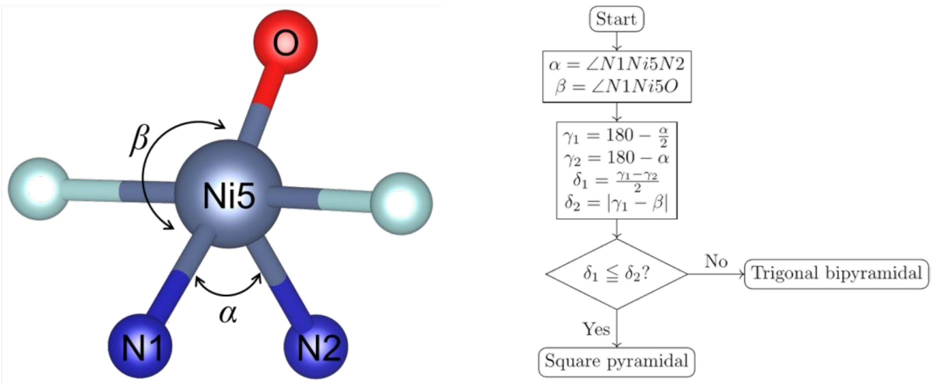

Figure S7. Flowchart to identify the coordination configuration of Ni[5] with the adsorbed species after geometry optimization.

9. Oxidation states assignment via magnetic moment

Table S3. Idealized magnetic moment approximated from net atomic spin projections.

M_{Ni} μ_B				M_{Fe} μ_B			
MO[a]	PBE	HSE $\alpha = 0.15$	HSE $\alpha = 0.25$	MO	PBE	HSE $\alpha = 0.15$	HSE $\alpha = 0.25$
2	1.3 – 1.8	1.4 – 1.9	1.5 – 1.9	5	3.9-4.2	3.9-4.2	3.9-4.2
1.5	1.2 – 1.3	1.3 – 1.4	1.2 – 1.5	--	--	--	--
1	0.6 – 1.2	0.6 – 1.3	0.8 – 1.2	--	--	--	--
0.5	0.3 – 0.6	0.3 – 0.6	0.3 – 0.8	--	--	--	--
0	0 – 0.3	0 – 0.3	0 – 0.3	--	--	--	--

[a] Idealized molecular orbital magnetic moment

10. Relative energy and magnetic moment for selected spin state

Table S4. The relative energies and magnetic moment for NiAl(100) and NiFe(100).

	Surface	NiAl (100)			NiFe (100)		
Function	Series	ΔE (eV)	$M_{Ni[1-6]}$	M_{total}	ΔE (eV)	$M_{FeNi[1-6]}$	M_{total}
PBE	S5	R	222002	7	1.24	5210.5220	12
	S4	0.68	222122	9	0.55	5212222	14
	S3	0.22	222221.5[a]	3	0.34	5222202	3
	S2	0.46	222220	2	0.21	5222222	17
	S1	0.00	222222	4	0.00	5222222	5
HSE α = 0.15	S5	2.06	222002	7	2.03	5220221	12
	S4	1.31	222122	9	1.24	5212222	14
	S3	0.99	222221.5	3	0.55	5222202	3
	S2	0.68	222220	2	0.06	5222222	17
	S1	0.00	222222	4	0.00	5222222	5
HSE α = 0.25	S5	2.40	222002	7	3.24	5210220	12
	S4	1.82	222122	9	1.75	5212222	14
	S3	1.52	222221.5	3	0.64	5222202	3
	S2	0.77	222220	2	0.05	5222222	17
	S1	0.00	222222	4	0.00	5222222	5

[a]The magnetic moment presented in the **Table S4** is idealized, approximated from the spin-density atomic projection according to **Table S3**.

Table S5. The relative energies and magnetic moment for *OH adsorbed NiAl(100) and NiFe(100).

	*OH	NiAl (100)				NiFe (100)			
Functional	Series	ΔE (eV)	$M_{Ni[1-6]}$	config	M_{total}	ΔE (eV)	$M_{FeNi[1-6]}$	config	M_{total}
PBE	S6	0.80	022222	bp	4	0.23	5222202	p	3
	S5	0.73	002212	p-H	1	0.30	5202212	p-H	4
	S4	0.37	022212	p-H	5	0.23	5222222	bp	12
	S3	0.32	202212	p-H	3	0.17	5222212	p	16
	S2	0.15	222222	bp	12	0.09	5222212	p-H	16
	S1	0.00	222212	p-H	11	0.00	5222212	p-H	6
HSE α = 0.25	S6	2.36	022222	bp	4	1.14	5222202	p	3
	S5	1.41	002212	p-H	1	0.64	5202212	p-H	4
	S4	0.76	022212	p-H	5	0.21	5222222	bp	12
	S3	0.65	202212	p-H	3	0.14	5222212	p	16
	S2	0.65	222222	bp	12	0.03	5222212	p-H	16
	S1	0.00	222212	p-H	11	0.00	5222212	p-H	6

Table S6. The relative energies and magnetic moment for *O adsorbed NiAl(100) and NiFe(100).

	*O	NiAl (100)				NiFe (100)			
Functional	Series	ΔE (eV)	$M_{Ni[1-6]}$	config	M_{total}	ΔE (eV)	$M_{FeNi[1-6]}$	config	M_{total}
PBE	S6	1.19	022012	p	1	0.41	5202222	bp	6
	S5	0.67	201.521.50.5	bp	8	0.24	5222222	bp	14
	S4	0.47	220.5212	p-H	6	0.26	5222222	bp	11
	S3	0.05	221.5221.5	bp	12	0.22	5222212	p	17
	S2	0.00	221.5222	bp	5	0.07	5222222	bp	3
	S1	0.00	222212	p-H	4	0.00	5222212	p	1
HSE α = 0.15	S6	1.89	022012	p	1	0.78	5202222	bp	6
	S5	1.53	20221.50	bp	8	0.31	5222222	bp	14
	S4	0.60	220212	p-H	6	0.17	5222222	bp	11
	S3	0.43	222222	bp	12	0.13	5222212	p	17
	S2	0.19	221.5222	bp	5	0.07	5222222	bp	3
	S1	0.00	222212	p-H	4	0.00	5222212	p	1
HSE α = 0.25	S6	2.24	02201.52	p	1	1.10	5202222	bp	6
	S5	1.94	20221.50	bp	8	0.47	5222222	bp	14
	S4	0.67	220212	p-H	6	0.03	5222222	bp	11
	S3	0.67	222222	bp	12	0.13	5222212	p	17
	S2	0.34	221.5222	bp	5	-0.05	5222222	bp	3
	S1	0.00	222212	p-H	4	0.00	5222212	p	1

Table S7. The relative energies and magnetic moment for *OOH adsorbed NiAl(100) and NiFe(100).

	*OOH	NiAl (100)				NiFe (100)			
Functional	Series	ΔE (eV)	$M_{Ni[1-6]}$	config	M_{total}	ΔE (eV)	$M_{FeNi[1-6]}$	config	M_{total}
PBE	S4	0.72	222211.5	bp	6	0.81	5222202	bp	11
	S3	0.56	222222	bp	8	0.41	5222222	bp	0
	S2	0.33	220.5212	p-H	3	0.21	5222212	p	16
	S1	0.00	222212	p-H	7	0.00	5222212	p	2
HSE α = 0.15	S4	1.49	222212	bp	6	1.46	5222202	bp	11
	S3	0.91	222222	bp	8	0.31	5222222	bp	0
	S2	0.65	220212	p-H	3	0.08	5222212	p	16
	S1	0.00	222212	p-H	7	0.00	5222212	p	2
HSE α = 0.25	S4	1.97	222212	bp	6	1.88	5222202	bp	11
	S3	1.15	222222	bp	8	0.22	5222222	bp	0
	S2	0.73	22021.52	p-H	3	0.05	522221.52	p	16
	S1	0.00	22221.52	p-H	7	0.00	522221.52	p	2

11. Electronic and geometric structure analysis

The energy profile of intermediates on NiAl/Fe are shown in **Figure S8-9**. For the NiAl/Fe surfaces, different color represents the parity of magnetic moment. And we can find that the even magnetic moment of NiAl surface obtains lower energy while the odd magnetic moment of NiFe surface tends to have lower energy, this is because the total number of valence charges of NiAl and NiFe has different parity. When the total magnetic moment of the system has the same parity as the number of total valence charges, the energy of the system tends to be lower, this is because different parity will result in fractional occupation. However, this result is at PZC condition, in the actual condition, the charge of each intermediate would not be zero and varied with the potential, therefore, the magnetic moment might be varied with potential as well and lead to spin crossover. For *OH adsorbed NiAl and NiFe, the pyramidal geometry configuration is more popular than bipyramidal configuration. Even we used the bipyramidal configuration initially, the final optimized geometry configuration will tend to be pyramidal configuration. This is mainly because of the PZC condition. According to the **Figure 4** and **S11**, we can find the stable geometry configuration varied with the external potential. For *O adsorbed surfaces, the occurrence possibility of two different geometry configurations is similar, although pyramidal configuration is a little bit more popular on *O/NiFe. For *OOH adsorbed surfaces, the pyramidal geometry configuration obtained lower energy compared to the bipyramidal one if the total magnetic moments are the same (if there is fractional occupation or spin flip in pyramidal configuration, the conclusion would be different). This is because the pyramidal geometry

configuration can form strong hydrogen bond which can lower the energy of system.

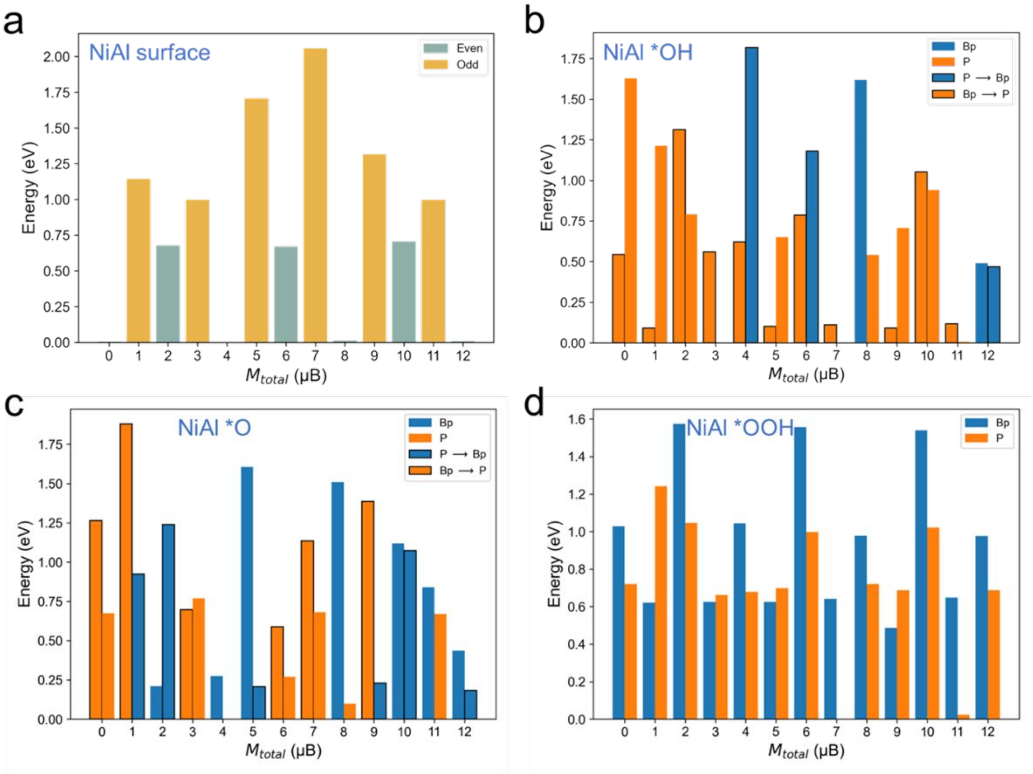

Figure S8. Energy profiles of intermediates on the NiAl surface: (a) pristine NiAl surface, (b) *OH adsorbed on NiAl, (c) *O adsorbed on NiAl, and (d) *OOH adsorbed on NiAl, considering various magnetic moments and geometric configurations.

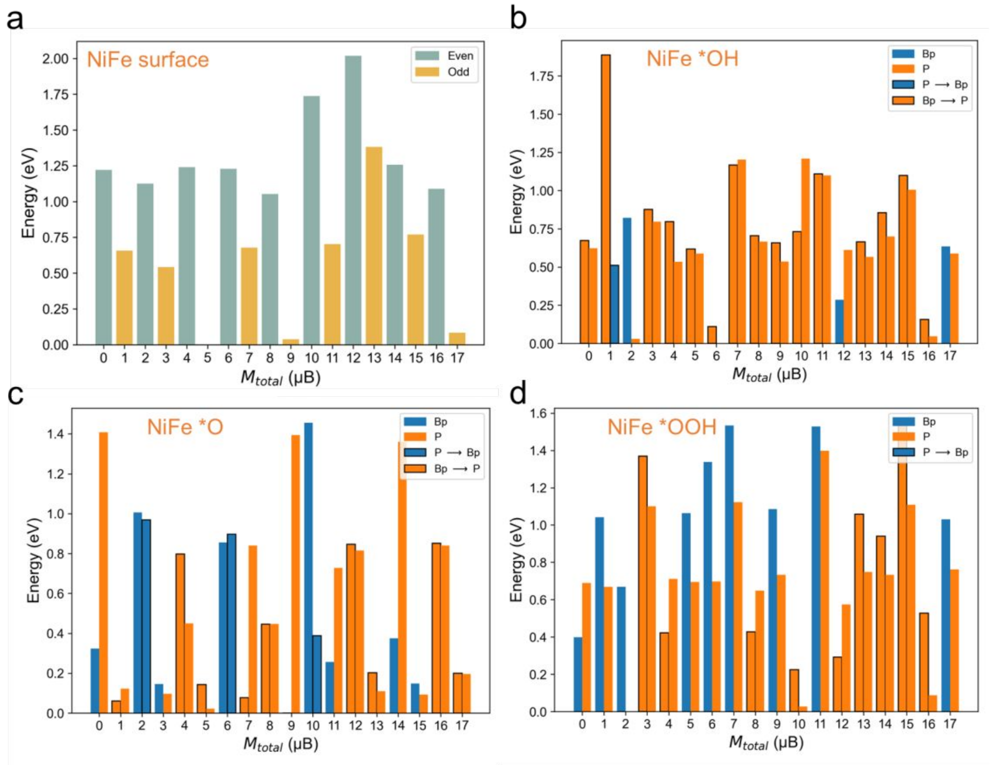

Figure S9. Energy profiles of intermediates on the NiFe surface: **(a)** pristine NiFe surface, **(b)** *OH adsorbed on NiFe, **(c)** *O adsorbed on NiFe, and **(d)** *OOH adsorbed on NiFe, considering various magnetic moments and geometric configurations.

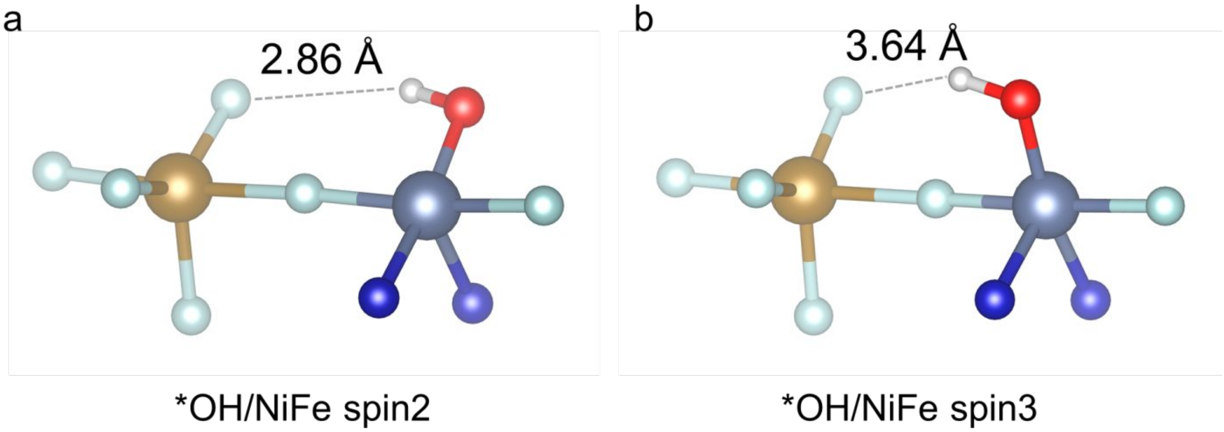

Figure S10. The structures of *OH/NiFe. **(a)** With and **(b)** without O-H···F hydrogen bond.

12. Spin-crossover under external potential

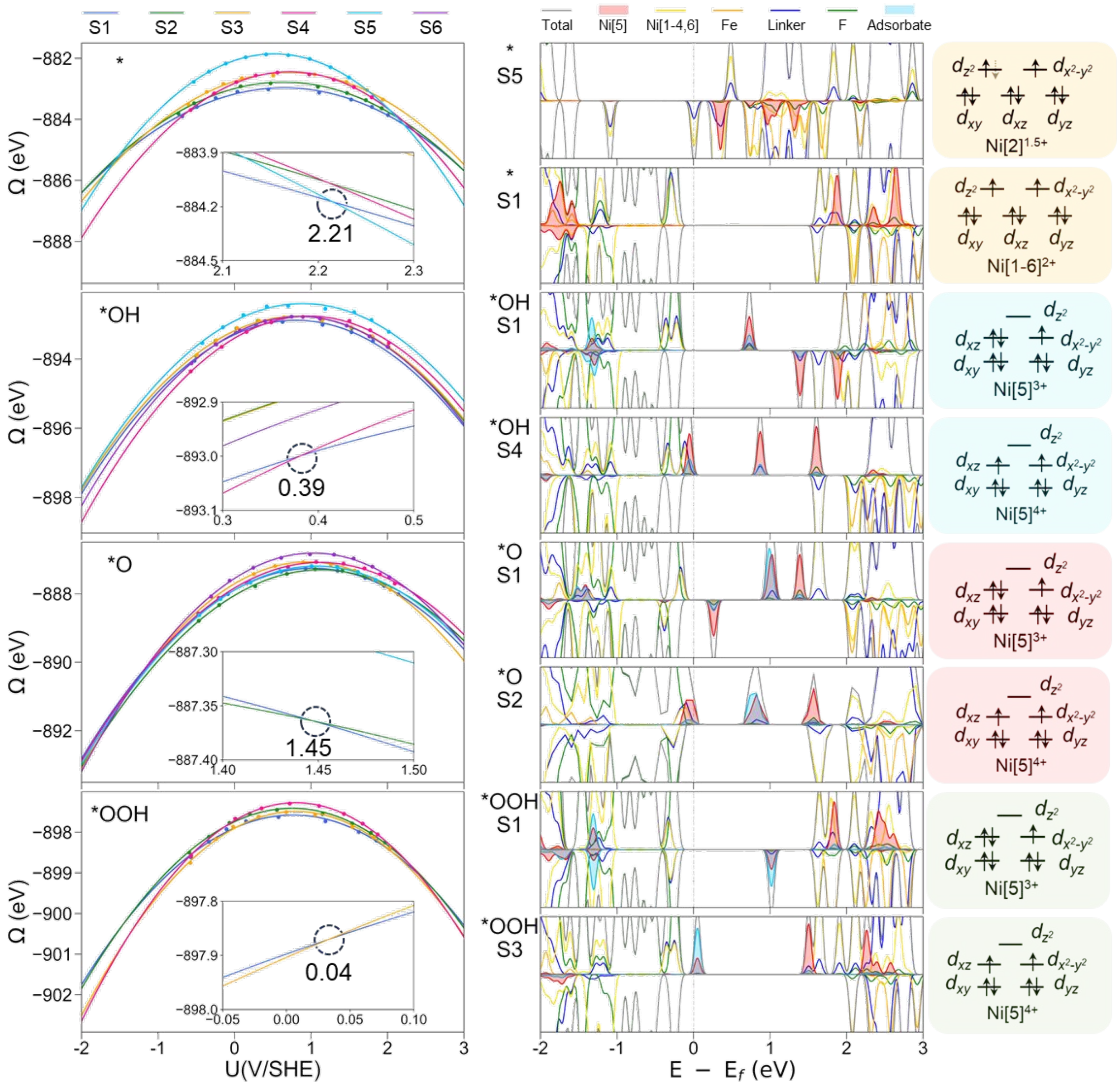

Figure S11. Calculated total energies (dots) and quadratic function fits (lines) of the selected six spin states of each intermediate **(a)** *, **(b)** *OH, **(c)** *O, and **(d)** *OOH on NiFe (100) surface as a function of external potential. The spin crossover points are circled by the dashed line, with the value of potential indicated underneath.

13. Spin density and DDEC charge

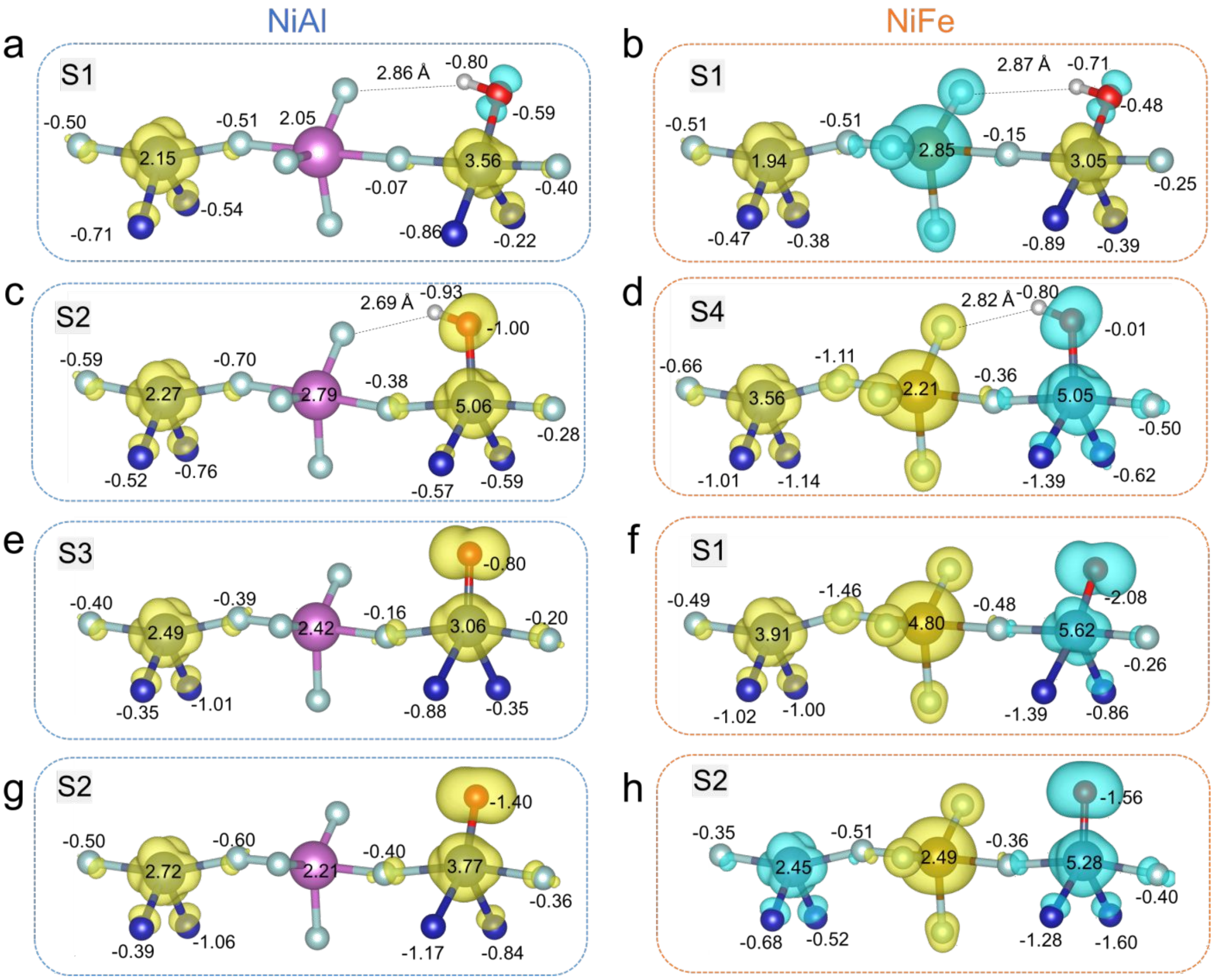

Figure S12. The spin density and DDEC charges for *OH/NiAl, *O/NiAl, *OH/NiFe and *O/NiFe. Yellow and blue represent the spin up and spin down density, respectively.

14. Reaction grand free energy and PDS

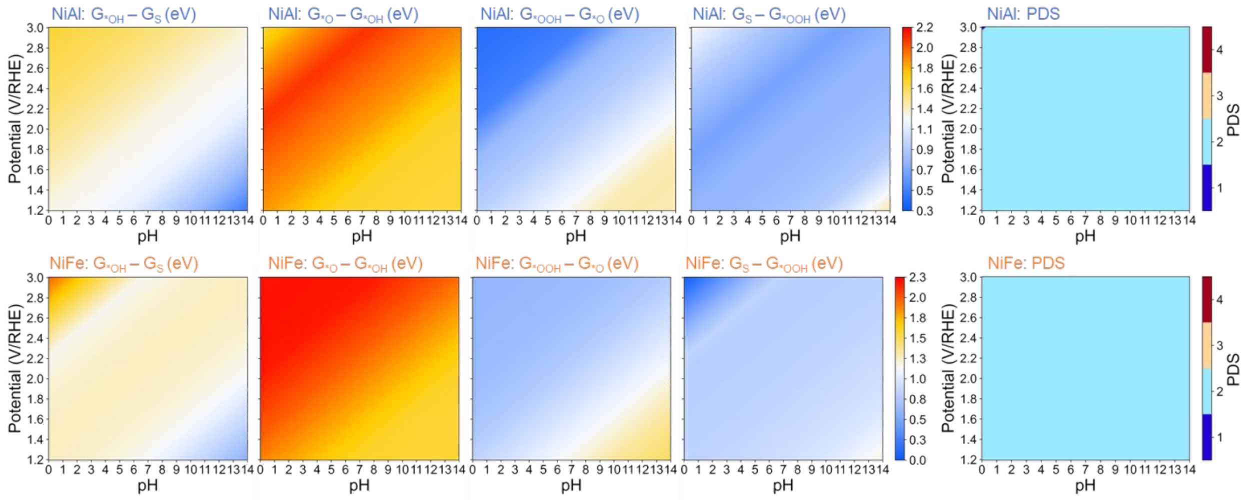

Figure S13. The reaction grand free energy and PDS for NiAl and NiFe under varied potential and pH conditions.

15. Fitted parameters of the quadratic equation

Table S8. Potential (U_0), energy (E_0), and the capacitances (C) of the selected spin states at PZC.

System		NiAl (100)			NiFe (100)		
Intermediate	Magnetic Order	U_0 (V/SHE)	C (e/V), (μF/cm^{-2})	E_0 (eV)	U_0 (V/SHE)	C (e/V), (μF/cm^{-2})	E_0 (eV)
*	S5	0.59	1.49, 15.81	-882.40	0.52	1.61,17.09	-881.86
	S4	0.69	1.08, 11.46	-883.11	0.72	1.47,15.56	-882.44
	S3	0.51	1.13, 12.01	-883.44	0.71	1.15,12.19	-882.46
	S2	0.44	1.16, 12.33	-883.15	0.64	1.04,11.02	-882.78
	S1	0.62	0.93, 9.82	-883.55	0.65	0.98,10.42	-882.97
*OH	S6	1.00	1.64, 17.42	-892.36	0.85	1.34,14.23	-892.78
	S5	0.88	1.46, 15.42	-892.46	0.89	1.27,13.43	-892.40
	S4	0.78	1.37, 14.53	-892.92	0.98	1.34,14.22	-892.76
	S3	0.88	1.45, 15.38	-892.90	0.82	1.26,13.31	-892.76
	S2	0.97	1.33, 14.08	-893.41	0.82	1.27,13.41	-892.77
	S1	0.81	1.25, 13.29	-893.48	0.81	1.27,13.46	-892.88
*O	S6	1.01	1.50, 15.91	-886.68	1.03	1.39,14.68	-886.81
	S5	0.89	1.53, 16.20	-887.09	1.07	1.23,13.02	-887.20
	S4	0.80	1.54, 16.36	-887.40	1.12	1.19,12.61	-887.09
	S3	1.00	1.22, 12.91	-887.96	0.96	1.38,14.67	-887.07
	S2	1.06	1.23, 13.06	-887.98	1.13	1.18,12.48	-887.30
	S1	1.00	1.38, 14.60	-887.77	1.03	1.21,12.84	-887.26
*OOH	S4	0.81	1.09, 11.56	-898.03	0.80	1.37,14.51	-897.27
	S3	0.81	1.56, 16.53	-897.98	0.81	1.27,13.48	-897.49
	S2	0.59	1.43, 15.19	-897.75	0.75	1.17,12.38	-897.41
	S1	0.74	1.20, 12.69	-898.11	0.77	1.09,11.53	-897.58

16. Computing the proportion of transfer coefficient

Herein, we follow the definition of transfer coefficient (α) in the book of Interfacial Electrochemistry,[11] in which the α can be derived from difference of $\Delta G^{\dagger}$ under varied external potential, and can be described as equation S12,

$$\Delta G_{ox}^{\dagger}(\phi) = \Delta G_{ox}^{\dagger}(\phi_0) - \alpha n F(\phi - \phi_0)\#(S12)$$

where $\Delta G_{ox}^{\dagger}(\phi)$ is the activation free energy of oxidation reaction under potential of ϕ, n is the number of transferred electrons, F is the Faraday coefficient.

To calculate the $\Delta G_{ox}^{\dagger}(\phi)$ and $\Delta G_{ox}^{\dagger}(\phi_0)$, we employed eNEB method developed by Duan et al which can be used to look for the transitional state under constant potential condition.[12] And we add $H_9O_4^+$ to the vacuum layer to simulate the solvent environment and to conduct the eNEB calculation. However, our calculation didn't find transitional states from the *OH to *O on NiAl (100) and NiFe (100) surfaces at potential of 0 V/SHE, even though we insert 5 points though IDPP method.[13] Therefore, we use reaction energy to represent the activation free energy instead (since the adsorbed species and explicit solvent environment for two surfaces are similar, we used ΔE rather than ΔG directly to save the computational resources). Another approximation is that we use the average of the number of transferred charges under different potential as the n. Since we only care about the trend of Tafel slope rather than the concrete value, the proportion of transfer coefficients of NiAl and NiFe are calculated. Related data are listed in Table S9.
The Tafel slope A is defined as

$$A = \frac{\lambda k_B T}{e\alpha}\#(S13)$$

where $\lambda = ln(10) = 2.30$, k_B is Boltzmann's constant, T is the absolute temperature, e is the electric elementary charge of an electron, α is the transfer coefficient. Therefore, the proportional of transfer coefficient for NiFe and NiAl can be calculated as equation S14,

$$\frac{\alpha_{NiFe}}{\alpha_{NiAl}} = \frac{A_{NiAl}}{A_{NiFe}}\#(S14)$$

where the value of proportion is 1.17. Theoretical value of it is 1.20.

Table S9. Energy data for the calculation of the proportion of transfer coefficient for *OH to *O.

		NiAl		NiFe	
	species	*OH	*O/TS[b]	*OH	*O/TS[b]
0 V/SHE [1.66 V/RHE]	Ω	-954.59	-953.58	-953.88	-952.87
	Ω(*O)-Ω(*OH)	1.01		1.01	
	$n(e^-)$	632.03	631.35	637.07	636.43
	$\Delta n(e^-)$	-0.67		-0.64	
1 V/SHE [2.66 V/RHE]	Ω	-953.94	-953.74	-953.24	-953.08
	Ω(TS)-Ω(*OH)	0.20		0.16	
	$n(e^-)$	630.78	630.00	635.76	635.13
	$\Delta n(e^-)$	-0.78		-0.62	
	ΔΩ(2.66V)-ΔΩ(1.66V)	-0.81		-0.85	
	$X[\Delta n(e^-)]$[a]	-0.73		-0.63	
Theoretical	α(NiFe)/α(NiAl)	1.20			
Experimental	α(NiFe)/α(NiAl)	1.17			

[a] The average of the number of transferred charges from *OH to *O.

b There is no transitional state (TS) at 0 V/SHE, so we use *O as the TS to calculate the activation grand energy. And TS is used to calculate activation grand energy at 1 V/SHE.

17. Effective mass at different K points.

The concept of effective mass is often employed as an indicator of electron and hole mobility within a material. Generally, a smaller absolute value of effective mass implies superior mobility. As indicated in Table S8, the effective mass of the NiFe material registers at -3.40 m* at T points, which is less than that of NiAl. Consequently, it can be deduced that the mobility of holes in NiFe bulk materials surpasses that in NiAl.

Table S10. Effective mass of holes in NiAl and NiFe bulk materials.

**Kpoints**	**X**	**Y**	**U**	**T**
NiAl	-3.73	-3.62	-3.74	-3.60
NiFe	-3.66	-3.71	-4.40	-3.40

18. Squared wavefunction analysis for NiAl and NiFe bulk materials

The conduction band minimum (CBM) and valence band maximum (VBM) of NiAl and NiFe bulk material are shown in **Figure S13-14**. From the **Figure S13 a-d**, we can find that the CBM and VBM of NiAl has overlap at N and Ni. Therefore, the Ni and N can serve of the center of recombination of hole and electron, which might hinder the separation of hole and electron, and lower the concentration of carriers. As a result, the lower concentration of carriers might lead to lower conductivity and bad OER performance.

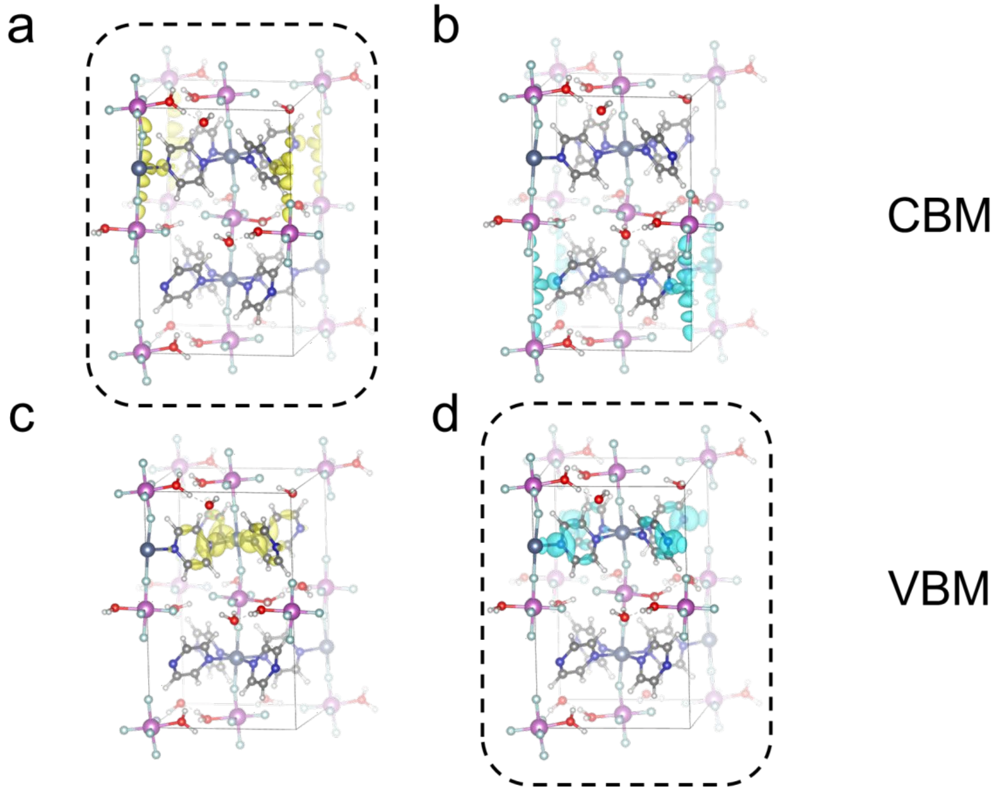

Figure S14. The squared wavefunction of NiAl at CBM and VBM. **(a, b)** CBM and **(c, d)** VBM. The yellow and blue region represent spin up and spin down density, respectively. Dashed black line circled **(a)** and **(d)**, this is because the CBM in **(a)** shared the same region with VBM in **(d)**, which will speed up recombination of holes and electrons.

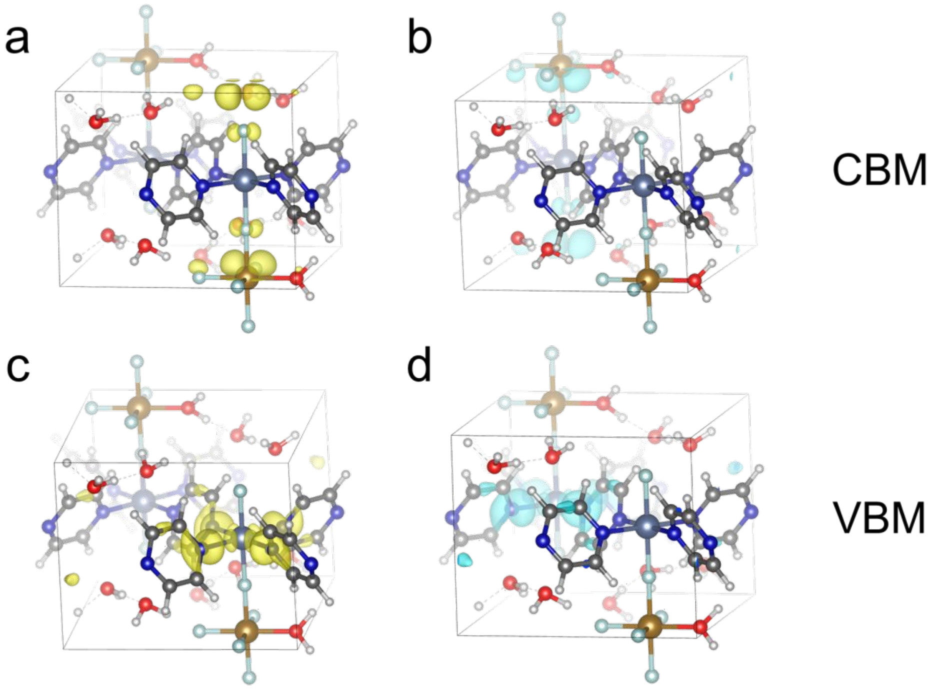

Figure S15. The squared wavefunction of NiFe at CBM and VBM. **(a, b)** CBM and **(c, d)** VBM. The yellow and blue region represent spin up and spin down respectively.

19. Future Investigation Guidance

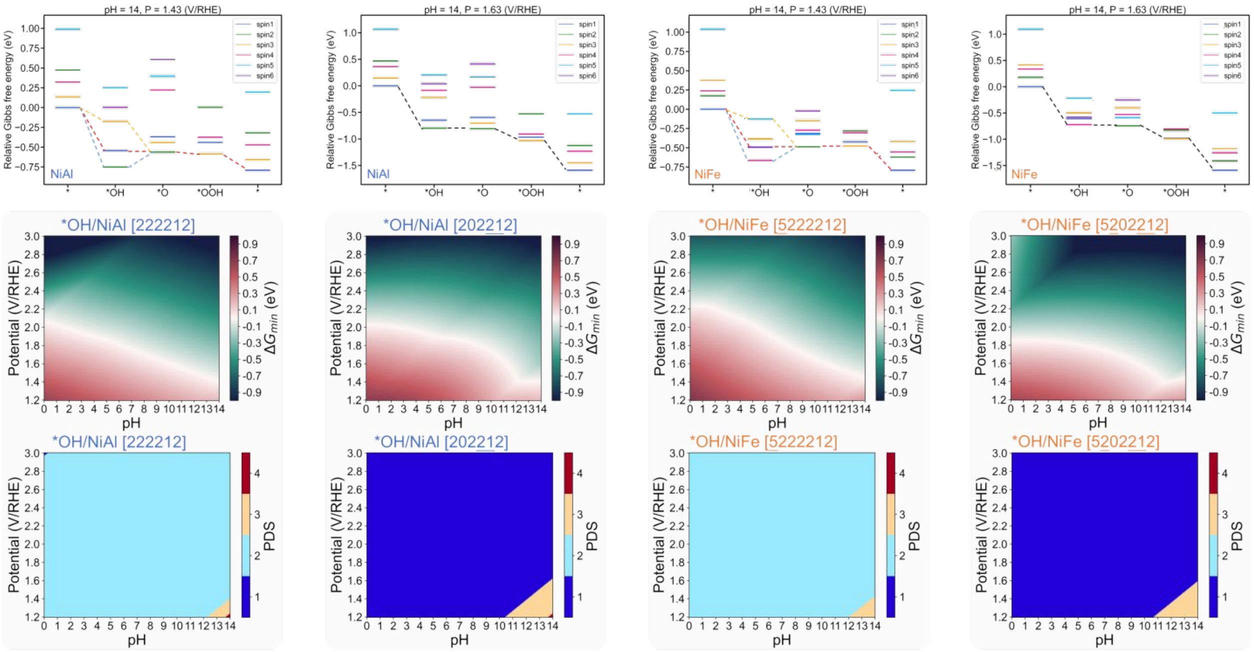

Figure S16. The free energy diagram of OER on the NiAl and NiFe surfaces under different external potentials at pH = 14. The ΔG_{min} and potential-determining step (PDS) of NiAl and

NiFe surfaces under varied external potential and pH, in which two different spin states of *OH intermediate are considered, respectively.

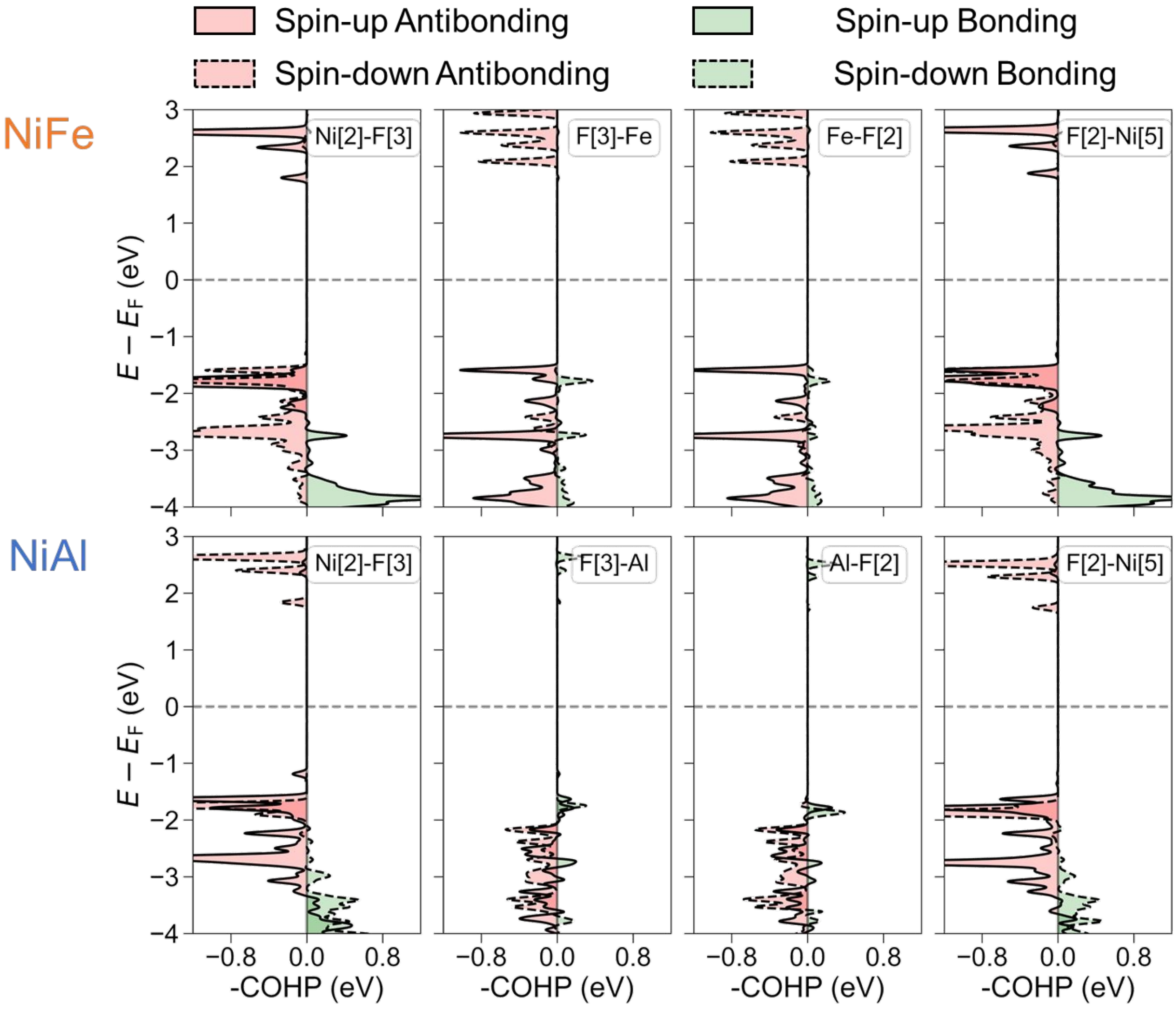

Figure S17. The COHP diagrams for the Ni[2]-F[3]-Fe/Al-F[2]-Ni[5] chains of the NiFe (100) and NiAl (100) surfaces, respectively. The spin state for NiFe (100) and NiAl (100) are S1 [5222222] and S1 [222222], respectively.

Table S11. The ICOHP for each component along the Ni-F-Al/Fe-F-Ni, O-Ni-F-Al/Fe and Ni-F-Al/Fe.

ICOHP		Surface			
Component		Ni[2]-F[3]	F[3]-Fe	Fe-F[2]	F[2]-Ni[5]
	Up	-2.24	-1.34	-1.33	-2.28
NiFe	Down	-1.84	-1.86	-1.85	-1.88
	Total	-4.08	**-3.21**	**-3.18**	-4.16
Component		Ni[2]-F[3]	F[3]-Al	Al-F[2]	F[2]-Ni[5]
	Up	-2.18	-3.76	-3.88	-2.03
NiAl	Down	-2.49	-3.59	-3.90	-2.48
	Total	-4.67	**-7.35**	**-7.78**	-4.51

The value of ICOHP reflects the bond strength: the more negative value, the stronger the bonding strength. The less native ICOHP values of Fe-F bonds suggest the weaker Fe-F interaction which is due to the more filled antibonding components in the valence band of Fe-F comparing to that of Al-F.

20. Experimental detail

a) Synthesis and Preparation

Synthesis of AlFFIVE-1-Ni (NiAl MOF)

Pyrazine (768.8 mg, 9.6 mmol), $Ni(NO_3)_2{\cdot}6H_2O$ (349.0 mg, 1.2 mmol), $Al(NO_3)_3{\cdot}9H_2O$ (450.0 mg, 1.2 mmol) and HF 48% (0.52 ml, 14.3 mmol) were mixed in a 20 mL Teflon liner. The mixture was diluted with 6 mL deionized water and then the autoclave was sealed and heated to 85 °C for 24 hrs. After cooling the reaction mixture to room temperature, the resultant blue-violet square-shaped crystals were collected by filtration and dried overnight at 70 °C.

Synthesis of FeFFIVE-1-Ni (NiFe MOF)

Pyrazine (768.8 mg, 9.6 mmol), $Ni(NO_3)_2{\cdot}6H_2O$ (349.0 mg, 1.2 mmol), $Fe(NO_3)_3{\cdot}9H_2O$ (484.8 mg, 1.2 mmol) and HF 48% (0.52 ml, 14.3 mmol) were mixed in a 20 mL Teflon liner. The mixture was diluted with 6 mL deionized water and then the autoclave was sealed and heated to 85 °C for 24 hrs. After cooling the reaction mixture to room temperature, the resultant orange-blue square-shaped crystals were collected by filtration and dried overnight at 70 °C.

b) Electrochemical Measurements

The electrochemical performance of the synthesized MOF electrocatalysts and commercial RuO_2 was conducted on a conventional three-electrode electrochemical cell (Autolab Nova Instruments). The three-electrode system was used with the prepared electrocatalysts supported on glassy carbon as the working electrode, a platinum wire electrode and an Ag/AgCl (saturated KCl) as the counter electrode and reference electrode, respectively. All the electrochemical

measurements were recorded in 1 M KOH (pH = 13.6) electrolytes and measured potentials were calibrated to reversible hydrogen electrode (RHE) via the following equation: $E_{(RHE)} = E_{(Ag/AgCl)} + 0.197 + 0.059pH$. Before electrochemical testing, high-purity N_2 gas was continuously bubbled through the electrolyte for over 10 mins to remove the dissolved O_2. The electrochemical accessibility of the working electrode was optimized by potential cycling between 1.10 and 1.65 V *vs.* RHE at scan rates of 20 mV s^{-1} in 1 M KOH electrolytes until stable voltammogram curves were obtained. In the OER measurements, linear sweep voltammetry (LSV) was performed at a scan rate of 5 mV s^{-1} for polarization curves and 2 mV s^{-1} for Tafel plots. All polarization curves of electrocatalysts in this work were corrected with 95% IR compensation.

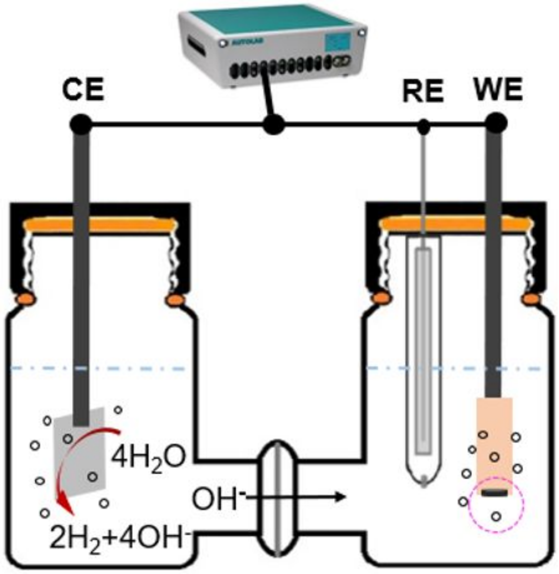

Figure S18. Configuration and set-up of a three-electrode electrochemical cell.

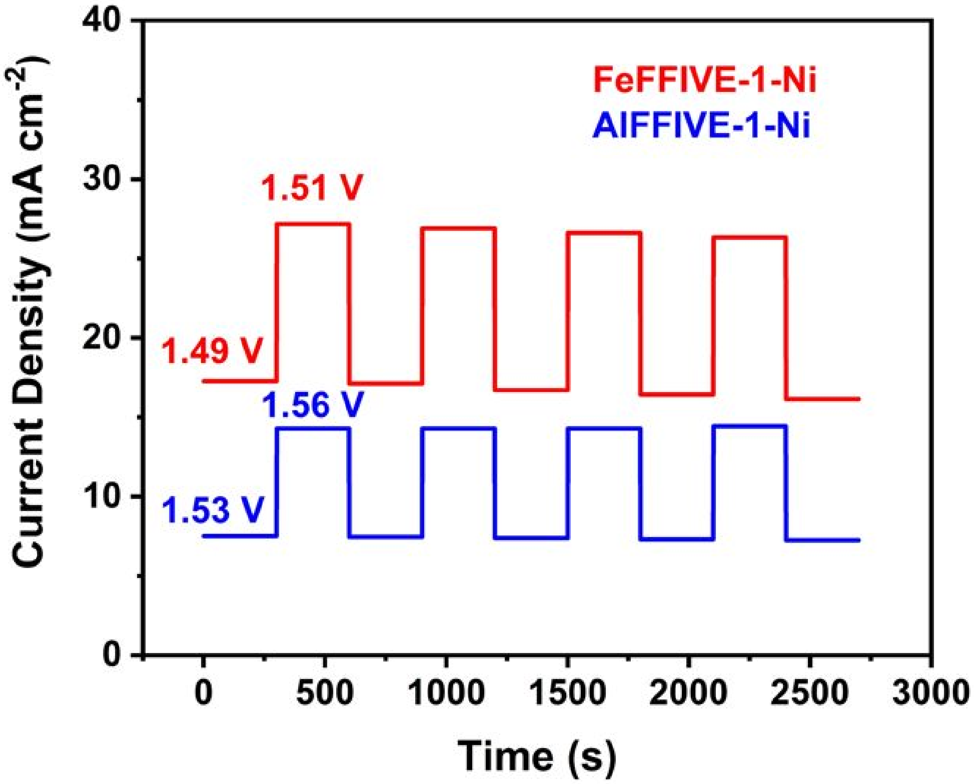

Figure S19. Cycling-response stability of NiAl and NiFe MOFs with changing constant voltages in 1 M KOH solution.

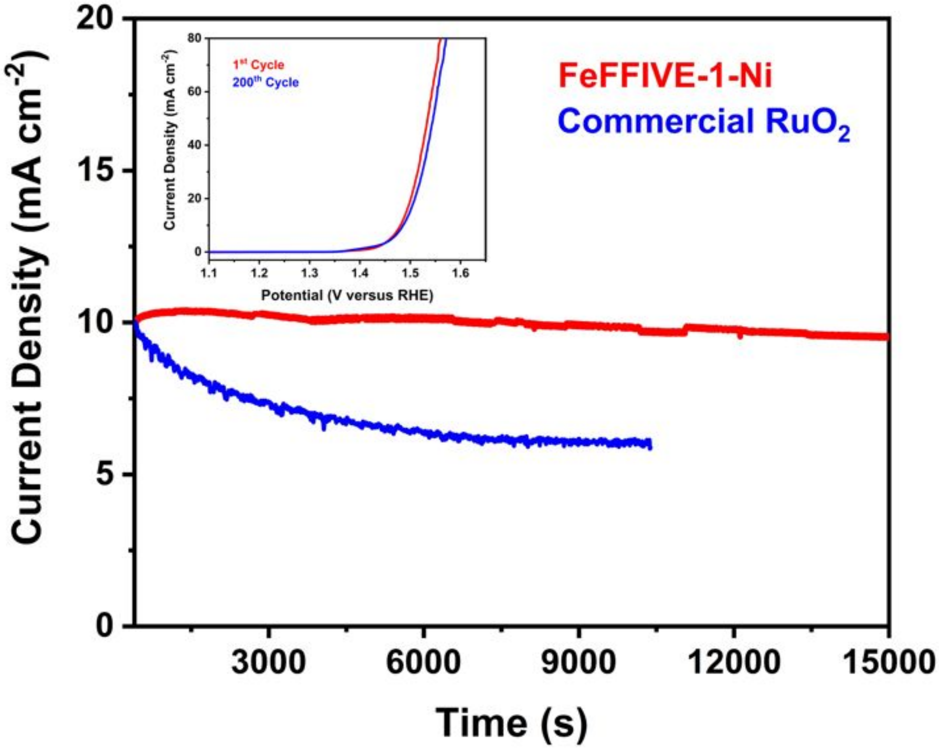

Figure S20. Long term stability of NiFe at constant overpotential of 253 mV with the comparison of commercial RuO_2 at constant 295 mV in 1 M KOH solution (inset: linear sweep voltammetry plots of the NiFe electrocatalyst obtained before and after 200 cycles).

Table S12. Summary of OER performance of MOF electrocatalyst in 1 M KOH electrolyte.

**MOF Electrocatalyst**	E_{onset} (V)	**Overpotential** (10 mA cm^{-2})	**Overpotential** (20 mA cm^{-2})	**Electrode Substrate**
NiAl	1.41	303 mV	337 mV	Glass Carbon
	--	250 mV	288 mV	Copper Foam
NiFe	1.36	253 mV	272 mV	Glass Carbon
	--	226 mV	262 mV	Copper Foam

c) Material Characterization

Powder X-ray diffraction (PXRD) patterns were recorded on a Bruker D8 Advance X-ray powder diffractometer with Cu Kα radiation (λ = 1.5418 Å) at a low scanning speed of 3° per min. Scanning electron microscopy (SEM) images were observed using a Magellan field emission scanning electron microscope at 5 kV with the software package for automated electron tomography. High-resolution transmission electron microscopy (HRTEM) was performed on the FEI Titan 60-300 electron microscope equipped with a spherical aberration (Cs) corrector and Gatan K2 direct-detection camera operated in electron counting mode under 300 kV. HAADF-STEM images were obtained on the Cs-corrected FEI Titan G2 60–300 Microscope operated at

300 kV. Probe Cs corrector was applied to get better spatial resolution. Moreover, elemental mapping analysis was collected by the TEM equipped with an electron energy loss spectroscopy (EELS). The specific surface area was obtained using the Brunauer-Emmett-Teller (BET) method on a Micromeritics ASAP 2420 system. X-ray photoelectron spectroscopy (XPS) was conducted using a Kratos Axis Ultra DLD spectrometer equipped with a monochromatic Al Kα X-ray source (hν=1486.6 eV) operating at 150 W, a multi-channel plate, and a delay line detector under vacuum. Binding energies were referenced to the C1s peak (set at 284.8 eV) of the sp3 hybridized carbon.

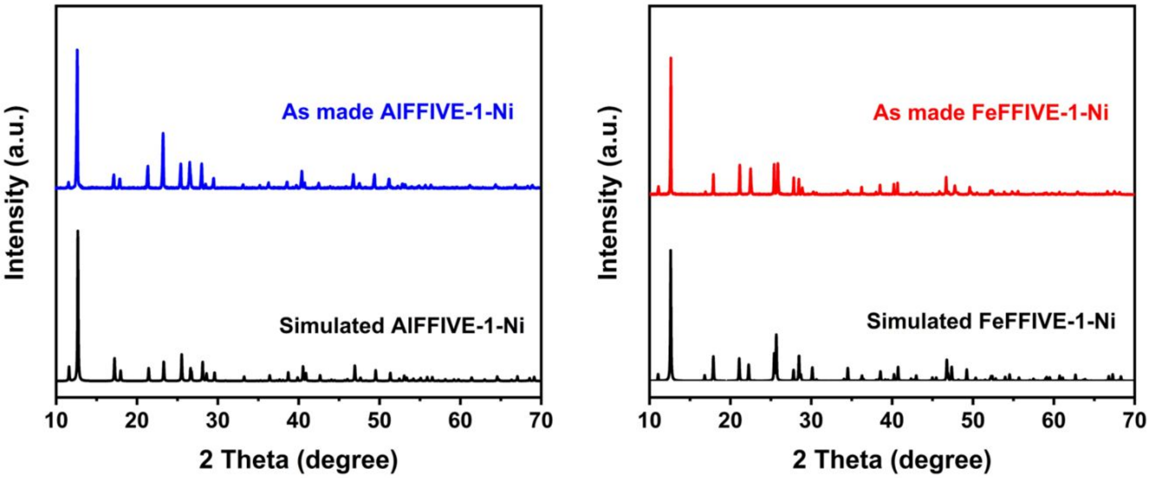

Figure S21. Powder X-ray diffraction (PXRD) patterns. (a) Simulated and experimental PXRD patterns of AlFFIVE-1-Ni. (b) Simulated and experimental PXRD patterns of FeFFIVE-1-Ni.

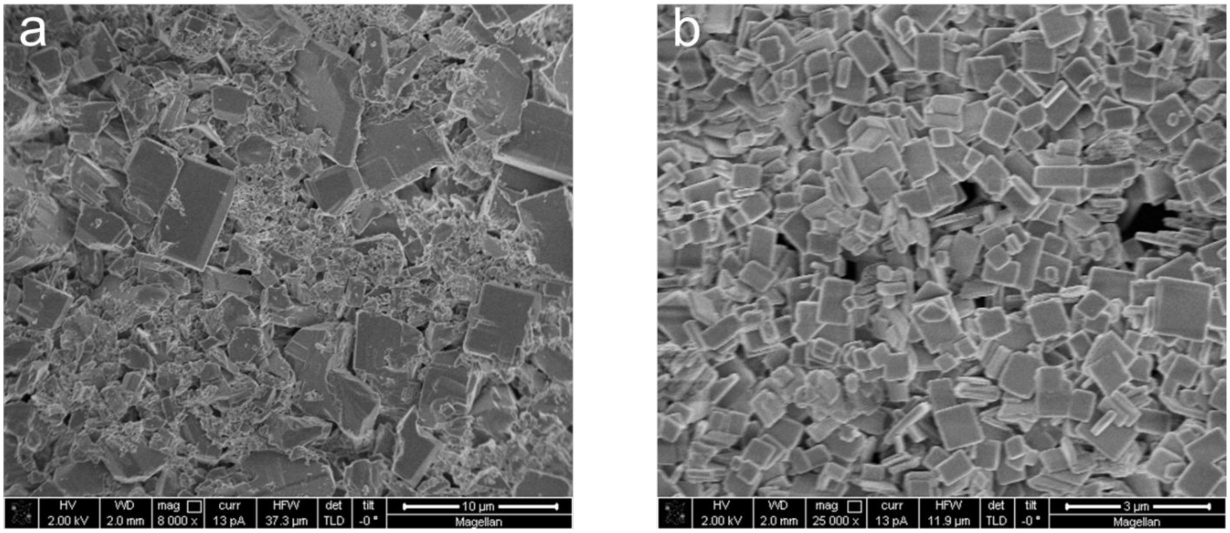

Figure S22. Scanning Electron Microscopy (SEM) images for (a) AlFFIVE-1-Ni and (b) FeFFIVE-1-Ni.

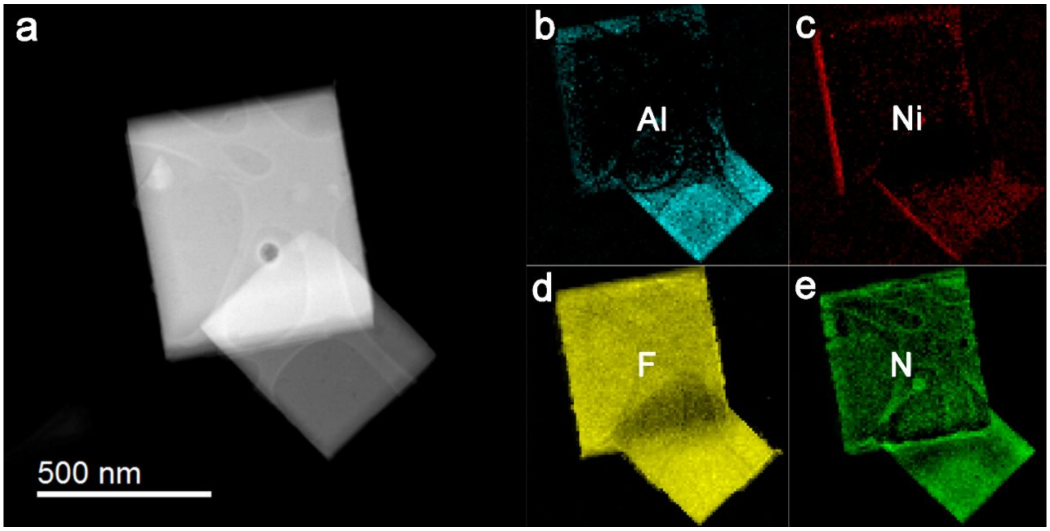

Figure S23. (a) HAADF-STEM image of AlFFIVE-1-Ni. (b-e) EELS-mapping of Al, Ni, F, and N elements, respectively.

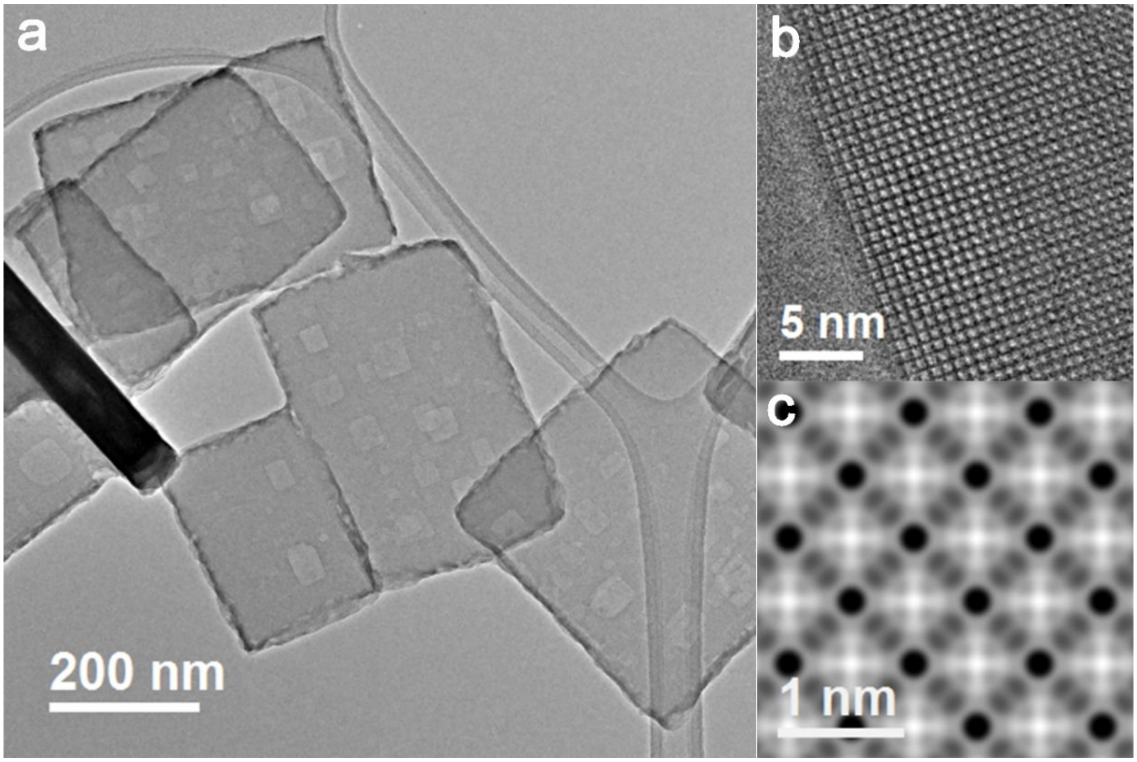

Figure S24. (a) TEM image of FeFFIVE-1-Ni. (b) HR-TEM image of FeFFIVE-1-Ni. (c) Modeled structure of FeFFIVE-1-Ni.

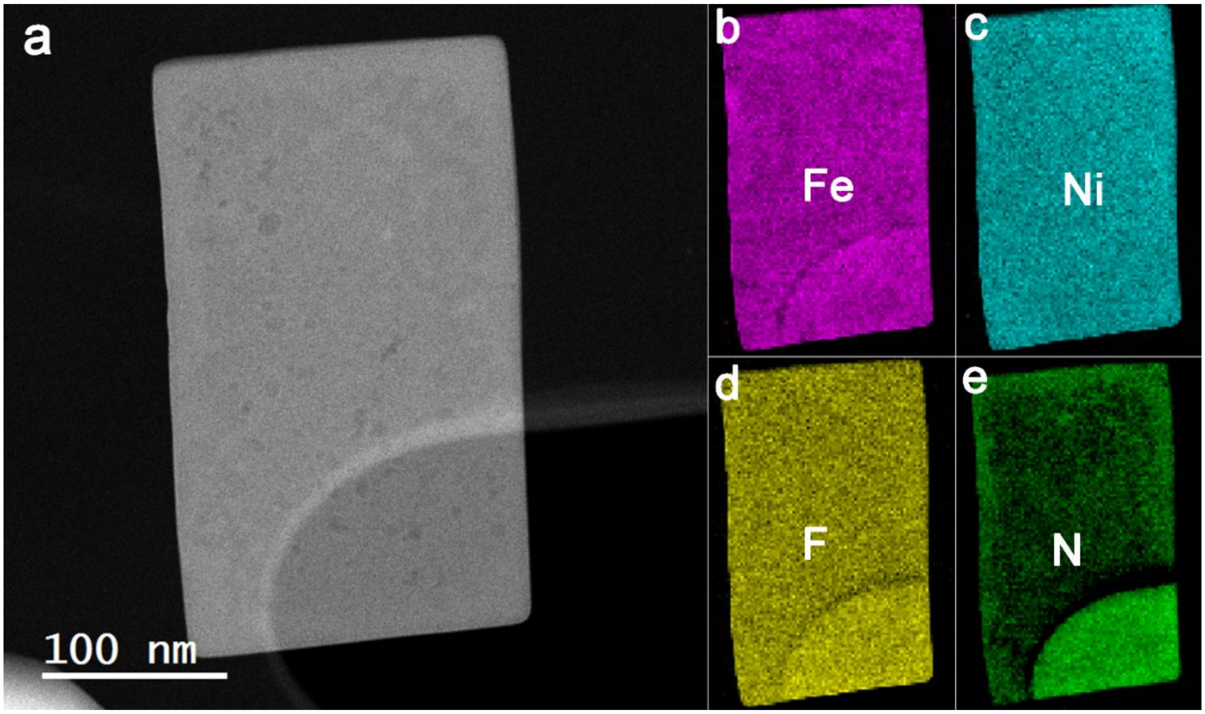

Figure S25. (a) HAADF-STEM image of FeFFIVE-1-Ni. (b-e) EELS-mapping of Fe, Ni, F, and N elements, respectively.

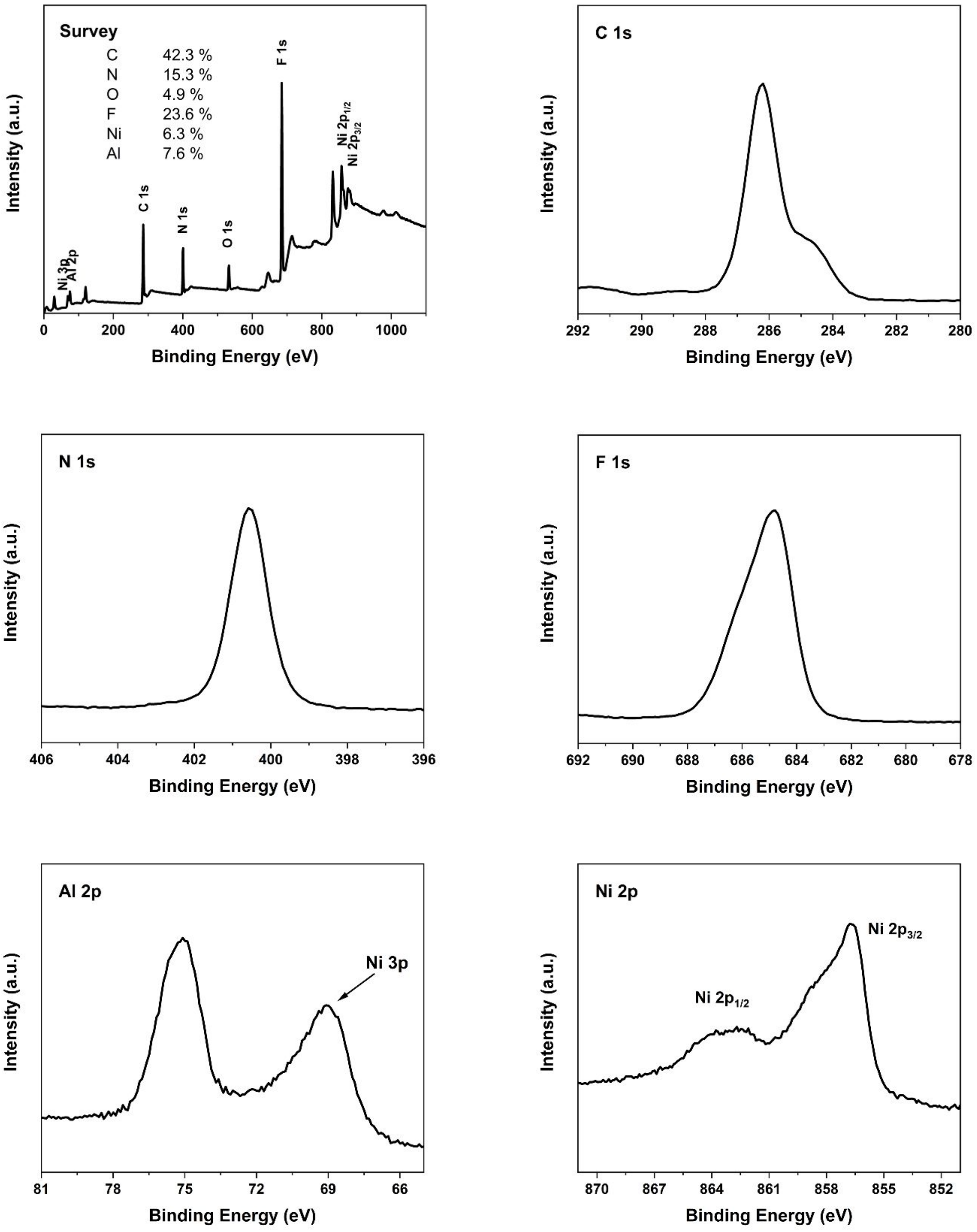

Figure. S26. XPS Characterization of AlFFIVE-1-Ni.

For an in-depth understanding of the atomic structure of AlFFIVE-1-Ni MOFs, X-ray photoelectron spectroscopy (XPS) can be employed. Two main peaks for Ni $2p_{3/2}$ and Ni 37 $2p_{1/2}$ located at 857 and 874 eV are accompanied by two shake-up satellite peaks (863.2 and 881.4

eV), respectively, which are characteristic features of Ni^{2+}. The Al 2p XPS of AlFFIVE-1-Ni has binding energies of 75.1 ($2p_{3/2}$) and 76.2 ($2p_{1/2}$) with a spin-energy separation of 1.1 eV, corresponding to the Al^{3+} phase.

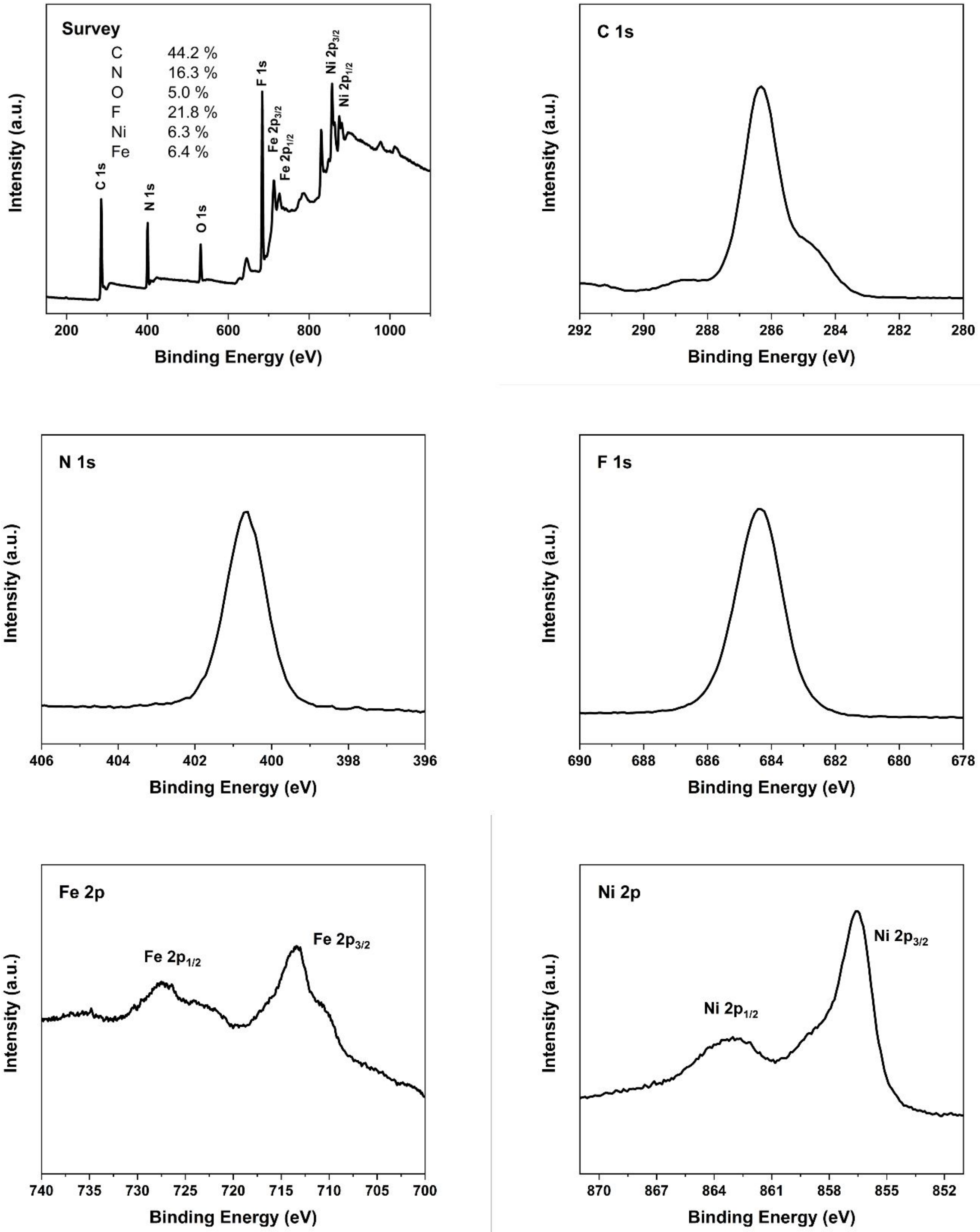

Figure. S27. XPS Characterization of FeFFIVE-1-Ni.

For the atomic structure of FeFFIVE-1-Ni MOFs, two main peaks for Ni $2p_{3/2}$ and Ni $2p_{1/2}$ located at 856.6 and 874.3 eV are accompanied by two shake-up satellite peaks (863.0 and 881.0 eV), respectively, which are characteristic features of Ni^{2+} . The Fe 2p XPS of FeFFIVE-1-Ni has binding energies of 713.4 ($2p_{3/2}$) and 727.5 ($2p_{1/2}$) with a spin-energy separation of 14.1 eV, corresponding to the Fe^{3+} phase.

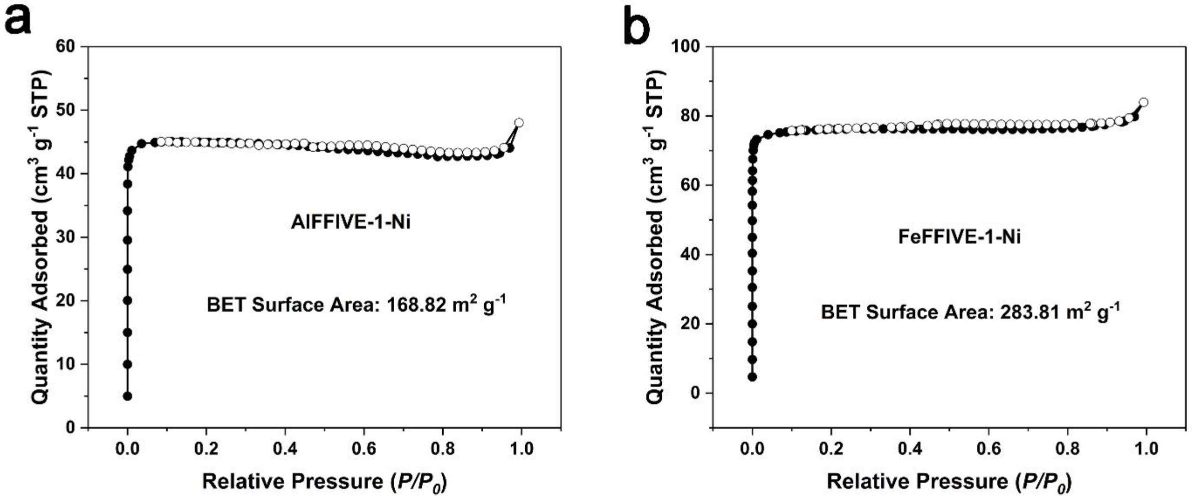

Figure S28. (a) N_2 adsorption-desorption isotherms of AlFFIVE-1-Ni at 77 K. (b) N_2 adsorption-desorption isotherms of FeFFIVE-1-Ni at 77 K.

21. References

(1) Datta, S. J.; Mayoral, A.; Murthy Srivatsa Bettahalli, N.; Bhatt, P. M.; Karunakaran, M.; Carja, I. D.; Fan, D.; Graziane M. Mileo, P.; Semino, R.; Maurin, G.; Terasaki, O.; Eddaoudi, M. Rational Design of Mixed-Matrix Metal-Organic Framework Membranes for Molecular Separations. *Science* **2022**, *376* (6597), 1080–1087. DOI: 10.1126/science.abe0192.

(2) Beaurepaire, E. Ultrafast Spin Dynamics in Ferromagnetic Nickel. *Phys. Rev. Lett.* **1996**, *76* (22). DOI: 10.1103/PhysRevLett.76.4250.

(3) Nørskov, J. K.; Rossmeisl, J.; Logadottir, A.; Lindqvist, L.; Kitchin, J. R.; Bligaard, T.; Jónsson, H. Origin of the Overpotential for Oxygen Reduction at a Fuel-Cell Cathode. *J. Phys. Chem. B* **2004**, *108* (46), 17886–17892. DOI: 10.1021/jp047349j.

(4) Chase, M.; Davies, C.; Downey, J.; Frurip, D.; McDonald, R.; Syverud, A. NIST-JANAF Thermochemical Tables. NIST Stand. Ref. *Database* **1985**. DOI: 10.18434/T42S31.

(5) Rossmeisl, J.; Logadottir, A.; Nørskov, J. K. Electrolysis of Water on (Oxidized) Metal Surfaces. *Chem. Phys.* **2005**, *319* (1–3), 178–184. DOI: 10.1016/j.chemphys.2005.05.038.

(6) Rossmeisl, J.; Dimitrievski, K.; Siegbahn, P.; Nørskov, J. K. Comparing Electrochemical

and Biological Water Splitting. *J. Phys. Chem. C* **2007**, *111* (51), 18821–18823. DOI: 10.1021/jp077210j.
(7) Rossmeisl, J.; Qu, Z.-W.; Zhu, H.; Kroes, G.-J.; Nørskov, J. K. Electrolysis of Water on Oxide Surfaces. *J. Electroanal. Chem.* **2007**, *607* (1), 83–89. DOI: 10.1016/j.jelechem.2006.11.008.
(8) Man, I. C.; Su, H.-Y.; Calle-Vallejo, F.; Hansen, H. A.; Martínez, J. I.; Inoglu, N. G.; Kitchin, J.; Jaramillo, T. F.; Nørskov, J. K.; Rossmeisl, J. Universality in Oxygen Evolution Electrocatalysis on Oxide Surfaces. *ChemCatChem* **2011**, *3* (7), 1159–1165. DOI: 10.1002/cctc.201000397.
(9) Wang, V.; Xu, N.; Liu, J.-C.; Tang, G.; Geng, W.-T. VASPKIT: A User-Friendly Interface Facilitating High-Throughput Computing and Analysis Using VASP Code. *Comput. Phys. Commun.* **2021**, *267*, 108033. DOI: 10.1016/j.cpc.2021.108033.
(10) Ochterski, J. W. Thermochemistry in Gaussian. 19.
(11) Schmickler, W.; Santos, E. *Interfacial Electrochemistry*; Springer Berlin Heidelberg: Berlin, Heidelberg, 2010. DOI: 10.1007/978-3-642-04937-8.
(12) Duan, Z.; Xiao, P. Simulation of Potential-Dependent Activation Energies in Electrocatalysis: Mechanism of O–O Bond Formation on RuO_2. *J. Phys. Chem. C* **2021**, *125* (28), 15243–15250. DOI: 10.1021/acs.jpcc.1c02998.
(13) Smidstrup, S.; Pedersen, A.; Stokbro, K.; Jónsson, H. Improved Initial Guess for Minimum Energy Path Calculations. *J. Chem. Phys.* **2014**, *140* (21), 214106. DOI: 10.1063/1.4878664.
